# Anomalous phonon selection rules and Raman optical activity in RuO_2_

**DOI:** 10.1126/sciadv.aeh0887

**Published:** 2026-10-21

**Authors:** Di Cheng, Ao Wu, Hui Pan, Yu-Peng Zhu, Shizhuo Luo, King Yau Yip, Wei Zhang, Ariando Ariando, Chang Liu, Jian-Sheng Wang, Xinwei Li

**Affiliations:** ^1^ Department of Physics, National University of Singapore, Singapore 117551, Singapore.; ^2^ Department of Physics, State Key Laboratory of Quantum Functional Materials, and Guangdong Basic Research Center of Excellence for Quantum Science, Southern University of Science and Technology (SUSTech), Shenzhen, Guangdong 518055, China.

## Abstract

Breakdown of selection rules and emergence of optical activity in Raman spectroscopy typically signal symmetry-breaking events essential for understanding novel phases of solids. Here, we apply our recently developed rotational anisotropy Raman spectroscopy technique to single-crystal ruthenium dioxide—a compound of recent interest as a candidate workhorse material for altermagnetism yet whose magnetic ground state remains under intense debate. Our data reveal a salient breakdown of selection rules for zone-center phonons, accompanied by clear splitting in the angle-dependent polarimetry plots for the two opposite helicity-nonconserving circularly polarized scattering channels. These observations provide definitive evidence of a previously overlooked symmetry-breaking event, involving either spatial or time-reversal symmetry. In either scenario, the underlying mechanism is likely a bulk hidden order parameter, potentially involving multipole moments of Ruthenium(IV) set by the local crystal field environment. Our work demonstrates how subtle symmetry-breaking events can manifest through lattice dynamics and calls for renewed experimental and theoretical efforts to map the phase diagram of ruthenium dioxide as a system poised near quantum phase transition boundaries.

## INTRODUCTION

Selection rules in Raman spectroscopy provide a valuable guide to new physical phenomena in crystalline solids (*1*, *2*). Across a phase transition, the symmetry reduction that accompanies the emergence of an order parameter offers a unified perspective on the associated changes in charge, spin, and lattice degrees of freedom. This symmetry lowering can induce new collective excitations and reshape the eigenfunctions of native phonon, electronic, or magnetic modes, thereby activating otherwise forbidden modes in selected scattering configurations. Symmetry-breaking events and the ensuing reconstruction of selection rules remain a recurring theme in optical condensed matter physics, underpinning observations that range from routine identification of defect-, strain-, or surface-induced structural distortions (*3*–*7*) to the sensitive detection of exotic, order parameter–coupled quantum excitations in charge density wave (*8*, *9*), magnetic (*10*–*13*), and superconducting systems (*14*) and in materials with hidden order (*15*–*17*).

Nowhere is the interplay of symmetry, magnetism, and electronic structure more vigorously discussed than in rutile RuO_2_ (*18*), a material long exploited for electrocatalysis and energy storage (*19*) that has traditionally been regarded as a Pauli paramagnet. Recent neutron and resonant x-ray scattering experiments have reported evidence of native collinear antiferromagnetic order (*20*, *21*). This observation, combined with a spin group analysis incorporating both structural and magnetic symmetries, catalyzed the proposal of altermagnetism (*22*)—a class of magnets that “combines the best of both worlds” of conventional ferromagnets and antiferromagnets—with RuO_2_ emerging as a candidate workhorse material. While a variety of novel phenomena have been reported in support of altermagnetism in RuO_2_, the most recent reexaminations—notably μSR (*23*, *24*), neutron diffraction (*25*), and photoemission measurements (*26*)—suggest that pristine RuO_2_, particularly in single-crystal form, does not host long-range magnetic order and instead attribute a portion of earlier observations to experimental artifacts. The magnetic ground state of RuO_2_ is thus currently under intense debate.

One recognized reason for the controversy surrounding magnetism in RuO_2_ is the material’s sensitivity to various intrinsic and extrinsic factors, including intraorbital Coulomb correlation, spin-orbit coupling, strain, hole doping, and impurities (*27*, *28*). This sensitivity underscores its proximity to several quantum phase transition boundaries. Its electronic structure features Dirac nodal lines near the Fermi surface (*29*, *30*), which can lead to density-wave and Pomeranchuk instabilities in the spin channel, rendering the system intrinsically prone to phase transitions—of which altermagnetism may be only one possibility. Moreover, in light of the diverse unusual experimental phenomena that continue to be reported (*31*–*36*)—including transport properties and spintronic effects such as spin-splitter torques—there is an urgent need to clarify the ground-state symmetry properties of RuO_2_. The key question is whether these phenomena arise from fragile altermagnetism, stabilized only in specific samples, or whether a more general underlying framework can account for them without invoking altermagnetism.

Here, we report a salient breakdown of Raman selection rules for zone-center phonons in single-crystal RuO_2_, using a high-quality pristine sample in which no signature of altermagnetic ordering was detected. This anomalous selection rule was uncovered through our developed rotational anisotropy Raman spectroscopy (RA-Raman) scheme (*37*)—a symmetry-sensitive technique capable of determining full (rather than partial) Raman tensors—and cannot be accounted for by the reported structural point group of the paramagnetic phase. In circularly polarized scattering geometries, the phonons exhibit intensity pattern splitting in helicity-nonconserving channels, yielding a Raman optical activity (ROA) signal that definitively points to symmetry breaking overlooked in previous studies. After ruling out trivial explanations, we discuss potential origins of this observation, with emphasis on the possibility of hidden order parameters—potentially of multipolar nature—that break either spatial symmetry or time-reversal symmetry.

## RESULTS

### Sample and RA-Raman measurements

As shown in Fig. 1A, RuO_2_ crystallizes in a rutile structure ($P4_{2}/\mathrm{mnm}$ space group and $D_{4h}$ point group). The unit cell is tetragonal, with the crystal *c* axis—coinciding with the fourfold screw axis—being shorter than the *a* and *b* lattice vectors. Each Ru atom is octahedrally coordinated by six O atoms; these octahedra form an edge-sharing network along the *c* axis and a corner-sharing network in the *ab* plane. The two Ru sublattices are related by a nonsymmorphic fourfold screw rotation, a symmetry operation central to the alternating spin polarization in the theoretically predicted altermagnetic phase.

**Fig. 1.**
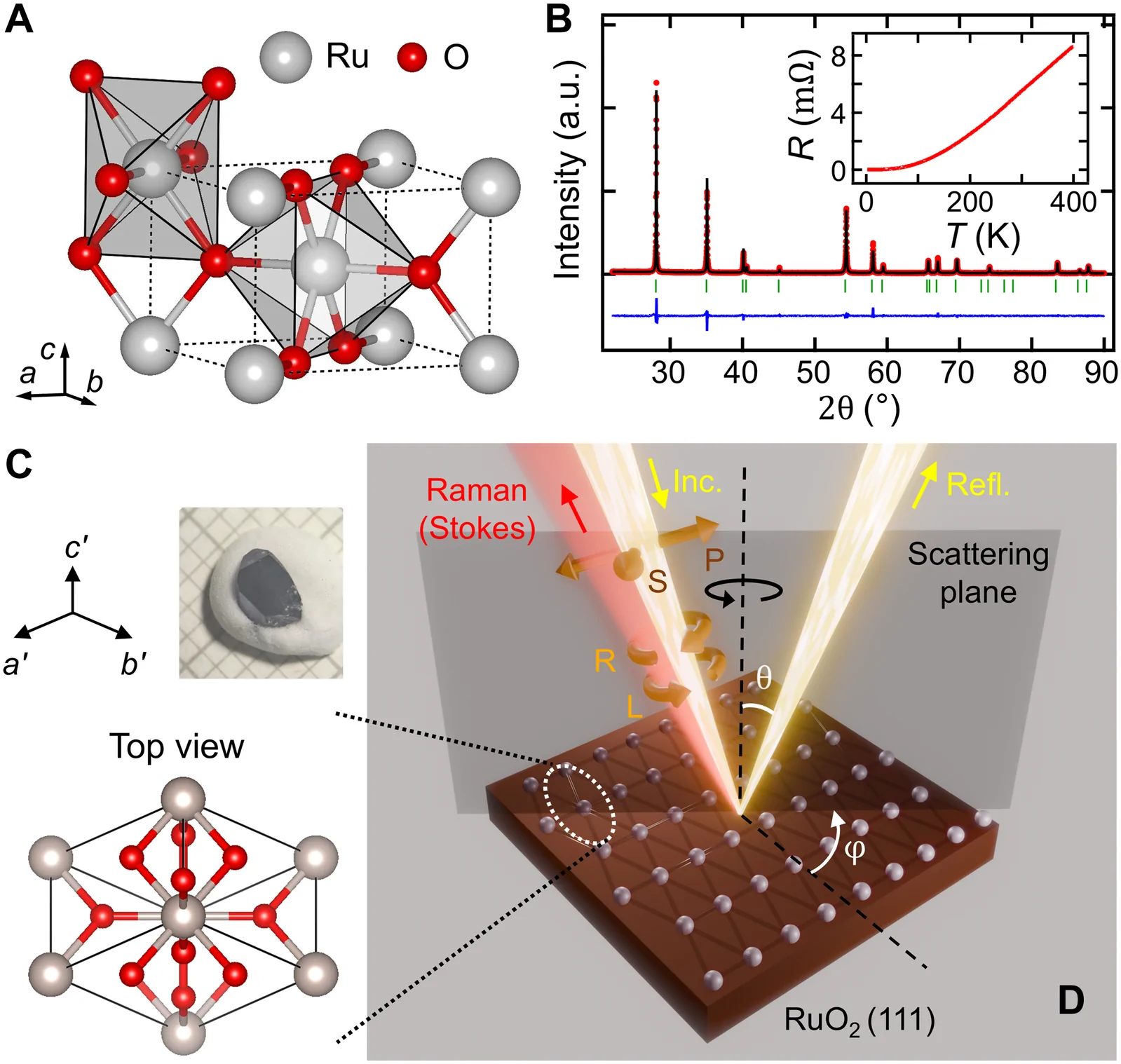
RuO_2_ sample and RA-Raman measurement geometry. (**A**) Crystal structure of RuO_2_. (**B**) Powder x-ray diffraction data and Rietveld refinement. Inset: Temperature-dependent electrical resistance of the single crystal. (**C**) Photograph of the RuO_2_ (111) single crystal and top-down view of the atomic structure. (**D**) RA-Raman measurement geometry: Backscattered intensity is recorded as a function of the scattering plane azimuthal angle φ, while the oblique incidence angle θ is held constant.

Our samples were synthesized by chemical vapor transport, wherein a mixture of polycrystalline RuO_2_ and TeCl_4_ powder was heated in a sealed silica tube. The resulting millimeter-size rod-like single crystals were partially ground into powder and characterized by powder x-ray diffraction (Fig. 1B). Rietveld refinement of the data using the GSAS-II software yielded a good fit to the Bragg peaks ($R_{\text{wp}}=10.53\%$), with no detectable impurity phases. Temperature-dependent electrical transport measurements (inset to Fig. 1B) recovered an ideal metallic response consistent with prior reports, exhibiting a residual resistivity ratio of 278—indicative of high sample quality. Crucially, systematic spin-resolved angle-resolved photoemission spectroscopy measurements performed on the same sample batch detected flat bands near the Fermi energy carrying spin polarization whose momentum-dependent texture aligns more closely with a surface-state description, while the bulk bands appear at odds with expectations for long-range magnetic order. The specific crystal used in our measurements is thus likely nonaltermagnetic.

For optical measurements, the crystal axes were oriented by Laue x-ray diffraction (fig. S1). The crystal (∼2 mm by 2 mm) was then cut along the [111] direction and polished to optical smoothness (Fig. 1C). Temperature control was achieved using a continuous-flow optical cryostat, in which the crystal was mounted and evacuated to a pressure below 2 × 10^−6^ torr.

All Raman measurements in this work were performed using our developed RA-Raman scheme (*37*). In contrast to conventional polarization-resolved Raman scattering (P-Raman), which fixes the photon wave vector while rotating the polarization, RA-Raman uses an oblique incidence geometry and measures scattering intensity during full azimuthal rotation of the optical scattering plane. As we have recently demonstrated (*37*), this scheme overcomes the inherent *C*_2_ constraint that limits all P-Raman measurements, enables complete determination of all Raman tensor elements, and substantially expands the accessible Fourier components in angle-dependent intensity patterns. Collectively, these capabilities yield markedly enhanced symmetry sensitivity.

As shown in Fig. 1D, an excitation laser beam (633 nm) is focused onto the RuO_2_ (111) sample at an oblique incidence angle θ = 10°. From the omnidirectional Raman scattering, we selectively collect the Stokes-shifted component that is strictly backscattered, retracing the path of the incident beam in the opposite direction (*37*). Anisotropy contrast is obtained by measuring the Raman intensity as a function of the azimuthal angle φ, defined as the rotation of the scattering plane—containing the incident beam and its specular reflection—about the sample’s surface normal. The incident polarization can be set to linear [s-polarized (S) or p-polarized (P)] or circular [right-circular (R) or left-circular (L)], with the analyzed polarization of the collected light independently configured for the same set of states (S, P, R, and L). Through a dedicated alignment procedure, the oblique incidence angle θ and polarization (defined with respect to the scattering plane) are precisely maintained, while φ is scanned, and the laser spot on the sample remains stationary throughout the measurement. This ensures that any anisotropy observed in the polar plots authentically reflects the true symmetry of the crystal lattice at the local focal spot, free from instrumental artifacts that would arise if the sample itself was rotated with stationary optical beams.

### Phonon selection rules and ROA

RA-Raman measurements were performed on (111)-cut RuO_2_ (Fig. 2A), with the corresponding φ-dependent Raman spectra acquired at *T* = 153 K shown in Fig. 2B. Three even-parity modes with distinct selection rules are observed, consistent with prior studies yet exhibiting substantially sharper linewidths (*38*–*40*): the doubly degenerate *E_g_* mode ($\omega_{E_{g}}=530\;{\text{cm}}^{-1}$), the $A_{1g}$ mode ($\omega_{A_{1g}}=649\;{\text{cm}}^{-1}$), and the $B_{2g}$ mode ($\omega_{B_{2g}}=720\;{\text{cm}}^{-1}$). Measurements were performed in four linear polarization configurations (PP, SS, PS, and SP) and four circular polarization configurations (RR, LL, RL, and LR), where the first and second letters denote the polarization of the incident and analyzed scattered light, respectively. For the PP and SS configurations, the spectral weight (*I*) of each mode was extracted by fitting the spectra to a sum of three Lorentzian peaks; the resulting φ dependence is presented as blue (PP) and red (SS) curves in Fig. 2 (C to E).

**Fig. 2.**
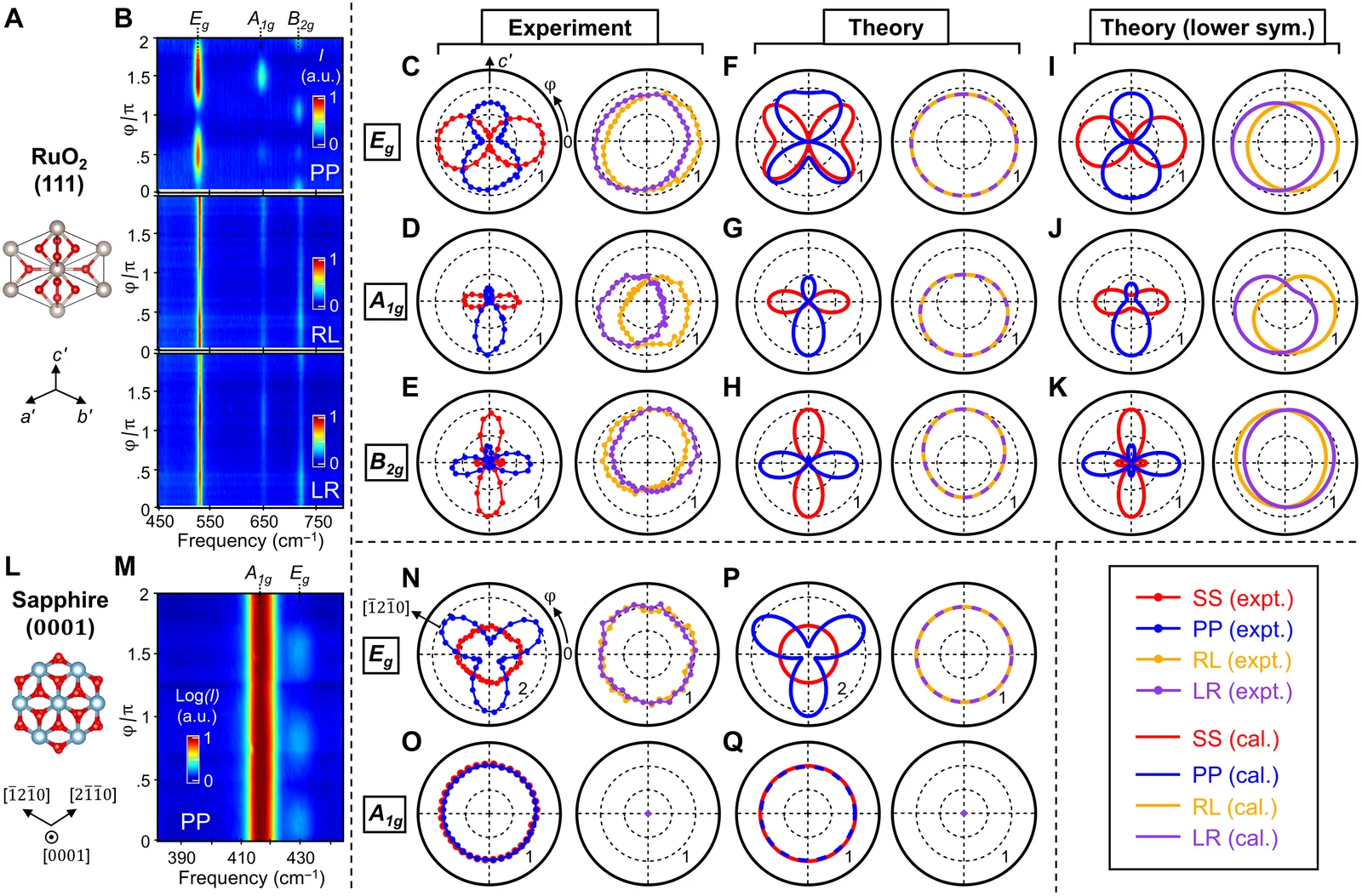
Experimental evidence for anomalous phonon selection rules and ROA in RuO_2_ (111). (**A** to **K**) Data and calculations for RuO_2_ (111). (**L** to **Q**) Data and calculations for sapphire (0001) as a benchmark sample. (A and L) In-plane crystal geometry. (B and M) Selected φ-dependent Raman spectra in the indicated polarization configurations. (C to E, N, and O) Experimental polar plots of φ-dependent intensities for various phonon modes in four polarization configurations (SS, PP, RL, and LR); these are compared with (F to H, P, and Q) Raman tensor theory assuming the reported crystallographic point group and (I to K) theory incorporating symmetry breaking. All measurements on RuO_2_ were performed at 153 K.

The polar plots $I_{\text{PP}}^{E_{g}}\left(\varphi \right)$, $I_{\text{PP}}^{A_{1g}}\left(\varphi \right)$, and $I_{\text{PP}}^{B_{2g}}\left(\varphi \right)$ all exhibit *C*_1_ symmetry—that is, no rotational symmetry. This behavior arises naturally from the tetragonal structure of RuO_2_, where ∣c∣<∣a∣=∣b∣. Consequently, the projections of the $\vec{a}$, $\vec{b}$, and $\vec{c}$ axes onto the (111) plane—denoted ${\vec{a}}^{\prime }$, ${\vec{b}}^{\prime }$, and ${\vec{c}}^{\prime }$—yield a distinct in-plane axis ${\vec{c}}^{\prime }$ (Fig. 1C). As determined by Laue x-ray diffraction, ${\vec{c}}^{\prime }$ is aligned along φ=90° in our polar plots, which also coincides with the mirror symmetry axis of all measured patterns, consistent with the crystal symmetry. These symmetry properties are revealed through the capabilities of the RA-Raman scheme, as conventional P-Raman measurements enforce identical geometries under a π rotation of the optical polarization, thereby imposing an artificial *C*_2_-symmetric pattern even for crystals that inherently lack it.

However, difficulties arise when interpreting $I_{\text{PP}}\left(\varphi \right)$ and $I_{\text{SS}}\left(\varphi \right)$ using the standard Raman theory

$$I\left(\varphi \right)\propto {|e_{\text{out}}^{†}\left(\varphi \right)\cdot R\cdot e_{\text{in}}\left(\varphi \right)|}^{2}$$ (1)

where **e**_in_ and **e**_out_ denote the incident and scattered polarization vectors, respectively, and the Raman tensor **R** is taken from standard tabulated results (discussed later) for the corresponding irreducible representation ($E_{g}$, $A_{1g}$, or $B_{2g}$) of the $D_{4h}$ point group. Treating the Raman tensor elements as complex-valued fitting parameters in an attempt to achieve the best possible agreement, we obtained poor fits between the theoretical polar plots (Fig. 2, F to H) and the experimental data (Fig. 2, C to E). Apparent discrepancies are evident for the $E_{g}$ mode, while the relative lobe intensities are misrepresented for both $I_{\text{PP}}^{A_{1g}}\left(\varphi \right)$ and $I_{\text{PP}}^{B_{2g}}\left(\varphi \right)$.

Even greater theory-experiment discrepancies emerge for measurements in circularly polarized configurations. Crucially, our oblique incidence geometry enables a φ-dependent investigation of these channels—a capability absent in conventional normal-incidence circular polarization experiments. The middle and bottom panels of Fig. 2B display the intensity maps in the RL and LR channels, from which the polar plots $I_{\text{RL}}\left(\varphi \right)$ (orange; Fig. 2, C to E) and $I_{\text{LR}}\left(\varphi \right)$ (purple; Fig. 2, C to E) are extracted. Notably, for all phonon modes, the $I_{\text{RL}}\left(\varphi \right)$ and $I_{\text{LR}}\left(\varphi \right)$ patterns differ—an unexpected observation, as contrast between helicity-nonconserving RL and LR channels, also known as ROA, typically signals either time-reversal symmetry (T) breaking or structural chirality (*41*, *42*). Neither condition is expected to hold in our RuO_2_ crystal. The Raman tensor theory (Eq. 1) for RuO_2_ (111), which accounts only for the crystallographic point group, predicts identical $I_{\text{RL}}\left(\varphi \right)$ and $I_{\text{LR}}\left(\varphi \right)$ (Fig. 2, F to H). This conclusion remains valid even when considering an accidental crystal miscut or complex-valued Raman tensor elements with arbitrary combinations of real and imaginary parts. This highlights the inadequacy of the current theoretical framework in explaining our experimental data and motivates a new model with reduced symmetry to fully and simultaneously account for the observations across all measurement channels (Fig. 2, I to K). Such a model will be discussed in Discussion.

To confirm that our observation of anomalous selection rules and ROA in RuO_2_ is physically meaningful, we performed comparative measurements on *c*-cut sapphire [α-Al_2_O_3_ (0001)] and (111)-cut Cd_2_Re_2_O_7_ crystals using the same RA-Raman setup. Sapphire crystallizes in the trigonal corundum structure with point group $D_{3d}$, for which the (0001) surface exhibits $C_{3}$ symmetry (Fig. 2L). Raman-active phonons transform as $A_{1g}$_-_ and $E_{g}$-irreducible representations (*43*), as shown in the spectral region highlighted in the intensity color map of Fig. 2M, with $\omega_{A_{1g}}=417\;{\text{cm}}^{-1}$ and $\omega_{E_{g}}=429\;{\text{cm}}^{-1}$. As shown in Fig. 2 (N and O), the extracted polar plot $I_{\text{PP}}^{E_{g}}\left(\varphi \right)$ exhibits *C*_3_ symmetry, faithfully reflecting the expected symmetry of sapphire (0001), while $I_{\text{SS}}^{E_{g}}\left(\varphi \right)$, $I_{\text{PP}}^{A_{1g}}\left(\varphi \right)$, and $I_{\text{SS}}^{A_{1g}}\left(\varphi \right)$ are all circularly symmetric. This behavior is fully captured by the nonmagnetic, achiral $D_{3d}$ structure of sapphire, as demonstrated by the theoretical fits in Fig. 2 (P and Q), which show excellent agreement with the experimental data. Moreover, the measured $I_{\text{RL}}\left(\varphi \right)$ and $I_{\text{LR}}\left(\varphi \right)$ perfectly overlap for both phonon modes, again consistent with the model’s expectations. The other control sample, Cd_2_Re_2_O_7_, is a semimetallic heavy-transition metal oxide known to undergo a cubic-to-tetragonal structural phase transition at cryogenic temperatures (*44*). Our RA-Raman measurements on this compound capture the expected symmetry evolution upon cooling (Supplementary Materials), and the phonon scattering intensity polar plots for the low-temperature tetragonal phase are precisely described by the standard Raman tensor theory, ruling out any experimental artifact specific to (111)-cut tetragonal systems. These measurements further highlight the unusual nature of the phonon selection rules and nonzero ROA in RuO_2_.

We then performed temperature-dependent RA-Raman measurements on RuO_2_ over the range of 103 to 523 K to track the evolution of the observed ROA. In the standard convention, ROA is defined as

$$\text{ROA}\left(\varphi \right)=\left[I_{\text{RL}}\left(\varphi \right)-I_{\text{LR}}\left(\varphi \right)\right]/\left[I_{\text{RL}}\left(\varphi \right)+I_{\text{LR}}\left(\varphi \right)\right]$$ (2)

from which we can construct a metric, $\int_{0}^{2\pi }|\text{ROA}\left(\varphi \right)|d\varphi$, which we denote as the integrated ROA, to quantitatively capture the visual deviation between the $I_{\text{RL}}\left(\varphi \right)$ and $I_{\text{LR}}\left(\varphi \right)$ polar plots. As shown in Fig. 3A, the integrated ROA decreases with increasing temperature, resembling the typical temperature evolution of an order parameter. However, complete suppression of this quantity is not achieved before visible surface degradation sets in at elevated temperatures. Representative polar plots of $I_{\text{RL}}\left(\varphi \right)$ and $I_{\text{LR}}\left(\varphi \right)$ for the $E_{g}$ and $A_{1g}$ modes at the lowest and highest measurement temperatures are shown in Fig. 3B. These plots demonstrate that the reduction in ROA at high temperatures indeed originates from a diminished overall discrepancy (highlighted by gray shading) between $I_{\text{RL}}\left(\varphi \right)$ and $I_{\text{LR}}\left(\varphi \right)$. We further fixed the measurement at the 153 K and performed a spatial scan across the sample. While spatial variations in ROA are observed, at no location did we detect a sign reversal—that is, an interchange of the $I_{\text{RL}}\left(\varphi \right)$ and $I_{\text{LR}}\left(\varphi \right)$ patterns—possibly due to pinning of order-parameter domains by extrinsic effects (*45*).

**Fig. 3.**
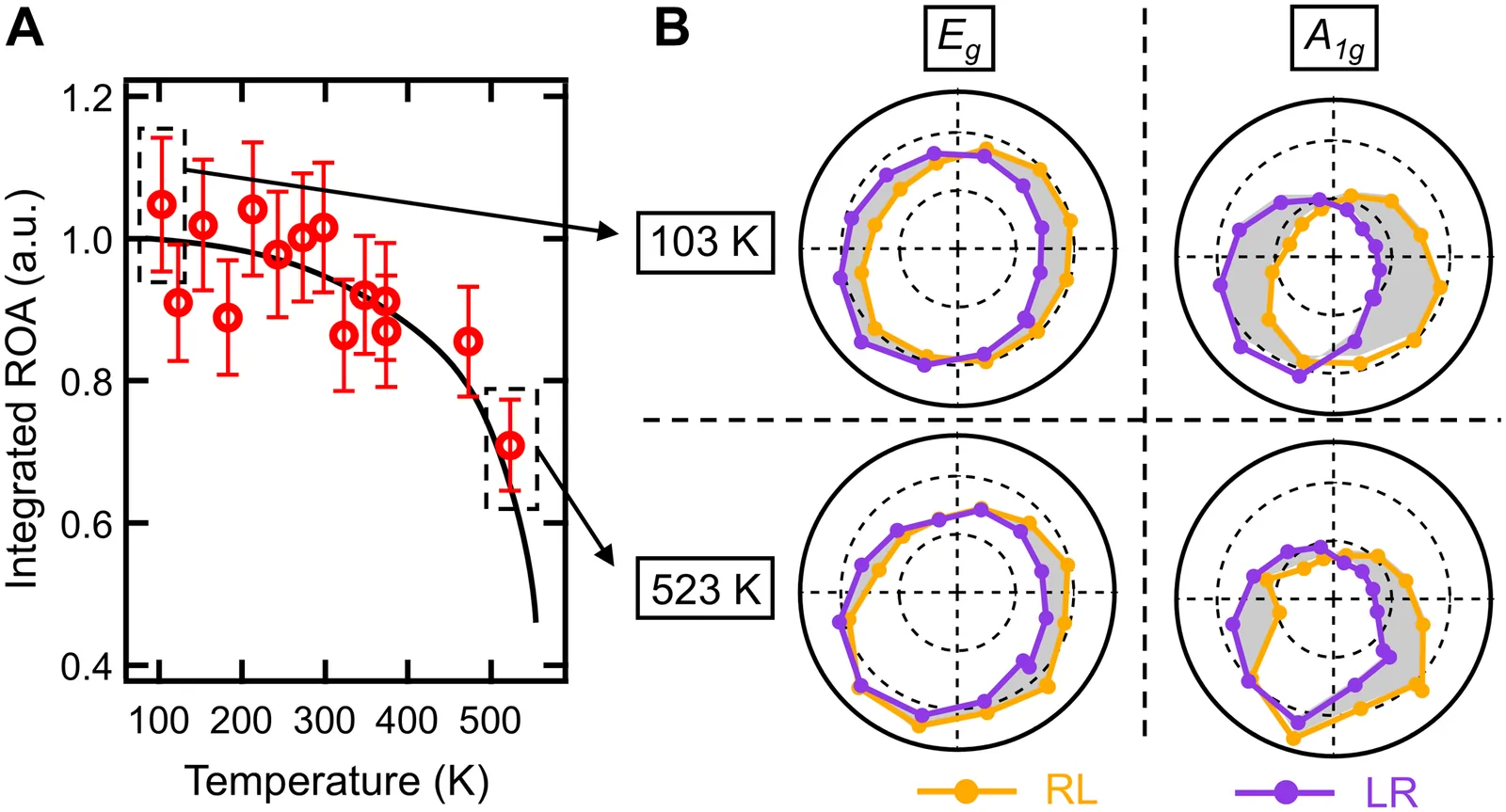
Temperature dependence of the ROA. (**A**) Integrated ROA associated with the $E_{g}$ mode versus temperature. (**B**) φ-Dependent intensities of $E_{g}$ and $A_{1g}$ phonon modes in RL and LR configurations at the highest and lowest measurement temperatures. The $I_{\text{RL}}-I_{\text{LR}}$ difference, highlighted by gray shading, is smaller at higher temperature.

## DISCUSSION

Before presenting a theory that incorporates additional symmetry breaking in RuO_2_, we address trivial alternative explanations for our observations. First, anomalous polarization selection rules and ROA can sometimes arise artifactually from optical birefringence (*46*), owing to polarization conversion as light propagates through an anisotropic crystal. However, reflectivity polarimetry measurements on our crystal—performed in the same incident geometry as the RA-Raman measurements—reveal negligible birefringence-induced polarization conversion (Supplementary Materials). This is because our probe photon energy (1.96 eV) lies in a nearly isotropic spectral region of the optical conductivity (*47*)—specifically, a crossover regime between intra-*t*_2g_ orbital transitions and the $t_{2g}\to e_{g}$ interband transitions. An electromagnetic wave model incorporating realistic measurement configurations, optical penetration depth, and the optical conductivity reported in prior ellipsometry studies fully corroborates this interpretation (Supplementary Materials).

Second, we considered the impact of finite wave vectors for Stokes-scattered phonons, which, in the backscattering geometry, equals twice the wave vector of the incident photon. In principle, the Raman tensor **R** can be modified by the phonon wave vector **k** as the mode deviates from the zone center, potentially relaxing selection rules. Since the magnitude of these wave vectors is small, it is typically sufficient to expand the **k** dependence to linear order

$$R\approx R^{0}+\overset{↔}{\chi }\cdot k$$ (3)

where **R**^0^ is the zone-center, **k**-independent part and $\overset{↔}{\chi }$ is a third-rank polar tensor with elements $\chi_{\alpha \beta \gamma }=\partial R_{\alpha \beta }/\partial k_{\gamma }$. Group theoretical arguments stipulate that for even-parity phonons in centrosymmetric crystals, all elements of $\overset{↔}{\chi }$ must identically vanish (*48*). The reported centrosymmetric $D_{4h}$ point group of RuO_2_ therefore dictates that finite wave vector effects do not alter the eigenfrequencies or eigenvectors to linear order for the Raman phonons reported here.

Third, resonant Raman scattering is also known to sometimes relax selection rules (*49*–*51*). When the incident photon is resonant with an intermediate electronic transition, the associated energy denominator in the Kramers-Heisenberg-Dirac formula becomes small, rendering the scattering process dominated by—and sensitive to—the symmetry of the intermediate electronic state. In certain cases, this can give rise to antisymmetric components in the Raman tensor (*50*), which may break selection rules and lead to optical activity. We ruled out this possibility through a thorough investigation based on first-principles calculations (Supplementary Materials). Our approach involves calculating phonon eigenvectors via density functional perturbation theory (DFPT), preceded by full cell relaxation. A nonmagnetic RuO_2_ unit cell was assumed, with relativistic spin-orbit coupling taken into account. Optical spectra were then computed, and the Raman tensor **R** was obtained by taking the derivative of the frequency-dependent dielectric function $\varepsilon_{\alpha \beta }\left(\omega ,Q\right)$ with respect to the distortion along the phonon eigenvector *Q*

$$R_{\alpha \beta }\left(\omega \right)\propto {\frac{\partial \varepsilon_{\alpha \beta }\left(\omega ,Q\right)}{\partial Q}|}_{Q=0}$$ (4)

a standard procedure for obtaining realistic Raman spectra that incorporate resonant effects (*52*). As detailed in the Supplementary Materials, the resulting Raman tensors for the $E_{g}$, $A_{1g}$, and $B_{2g}$ phonons at our probe photon energy (ℏω=1.96 eV) are generally complex valued but remain symmetric, satisfying $R_{\alpha \beta }=R_{\beta \alpha }$. The tensor structures fully retain the tabulated Raman tensor forms expected from standard group theory. This is illustrated in Fig. 4A, which displays the calculated Raman tensors; each cell shows the absolute magnitude of a tensor element, normalized by the largest element in its matrix. This conclusion holds regardless of whether spin-orbit coupling or a Hubbard *U* correction is included in the calculations. It also applies to a broad probe photon energy range (Supplementary Materials), potentially resonant with various interband transitions within the electronic structure, as revealed by our calculations—a prediction verifiable by future RA-Raman experiments using different probe laser wavelengths.

**Fig. 4.**
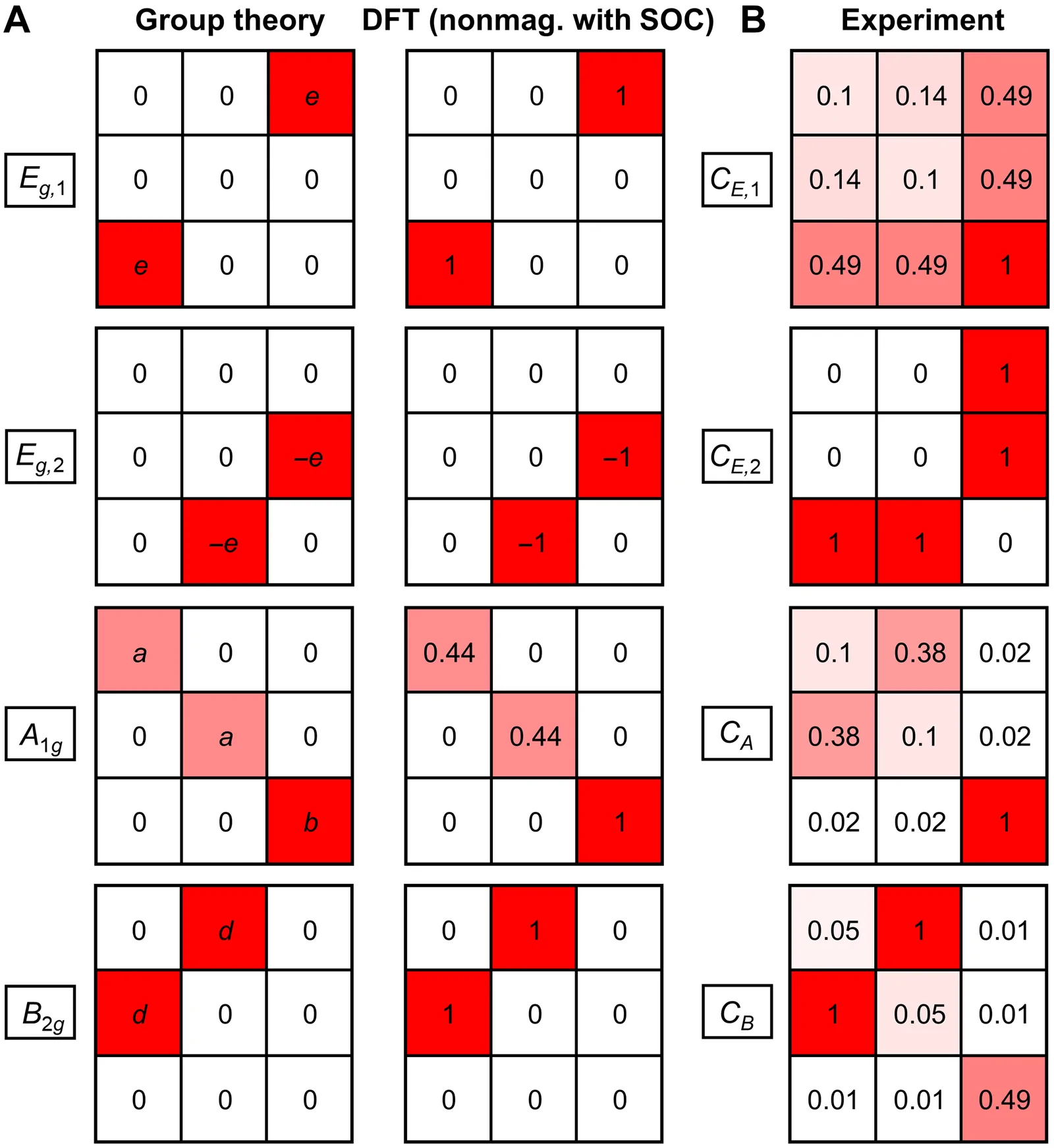
Comparison of Raman tensors between standard theory and experiments. (**A**) Raman tensors for the $E_{g}$, $A_{1g}$, and $B_{2g}$ phonons: Left panel shows tensors derived from group theory tables for the reported $D_{4h}$ point group; right panel shows tensors computed from first principles. (**B**) Raman tensors determined experimentally. Because of the observed mode mixing, the phonon labels are updated to $C_{E}$, $C_{A}$, and $C_{B}$ to reflect their mixed characters. For all tensors shown, each cell displays the absolute magnitude of a complex-valued tensor element, normalized by the largest element in its matrix.

The Raman tensors either computed from first principles or taken from tabulations for the $D_{4h}$ point group (Fig. 4A) stand in stark contrast to those determined experimentally (Fig. 4B). As demonstrated in prior work (*37*), the RA-Raman scheme enables full determination of Raman tensors, unlike conventional P-Raman setups, which detect only a subset of elements. This capability can be intuitively understood using a geometric analogy: A symmetric Raman tensor can be represented as a three-dimensional ellipsoid (or, more generally, a quadratic surface if not positive definite). While P-Raman captures only a single two-dimensional projection of this ellipsoid onto the plane perpendicular to the fixed wave vector, RA-Raman acquires a series of distinct two-dimensional sections by varying the orientation of the scattering plane, thereby fully constraining the ellipsoid’s geometry and enabling complete tensor reconstruction; to highlight this distinction, we performed incident angle–dependent measurements and identified key features in the RA-Raman data that are absent in normal-incidence P-Raman measurements (Supplementary Materials). Using the symmetry-breaking models described below, we derived the experimental Raman tensors displayed in Fig. 4B, which successfully reproduce all polar plot data across all eight measurement configurations (see partial plots in Fig. 2, I to K and a more extensive comparison in section S5). Substantial mixing between elements of the $E_{g}$, $A_{1g}$, and $B_{2g}$ modes is evident, indicating the need to go beyond the assumption of a nonmagnetic $D_{4h}$ structure.

Our interpretation of the observations is summarized schematically in Fig. 5A. The left column lists the zone-center phonon normal modes derived from the nonmagnetic reference structure with the $D_{4h}$ point group: the doubly degenerate $E_{g}$ mode, along with the $A_{1g}$ and $B_{2g}$ modes. As irreducible representations of $D_{4h}$, these modes are initially orthogonal to one another. However, upon symmetry lowering, this orthogonality is lifted, allowing the modes to hybridize and form a new manifold of mixed-character phonon modes (right column). These are labeled as $C_{E,1}$, $C_{E,2}$, $C_{A}$, and $C_{B}$, where the subscript denotes the dominant irreducible representation component inherited from the original $D_{4h}$ classification.

**Fig. 5.**
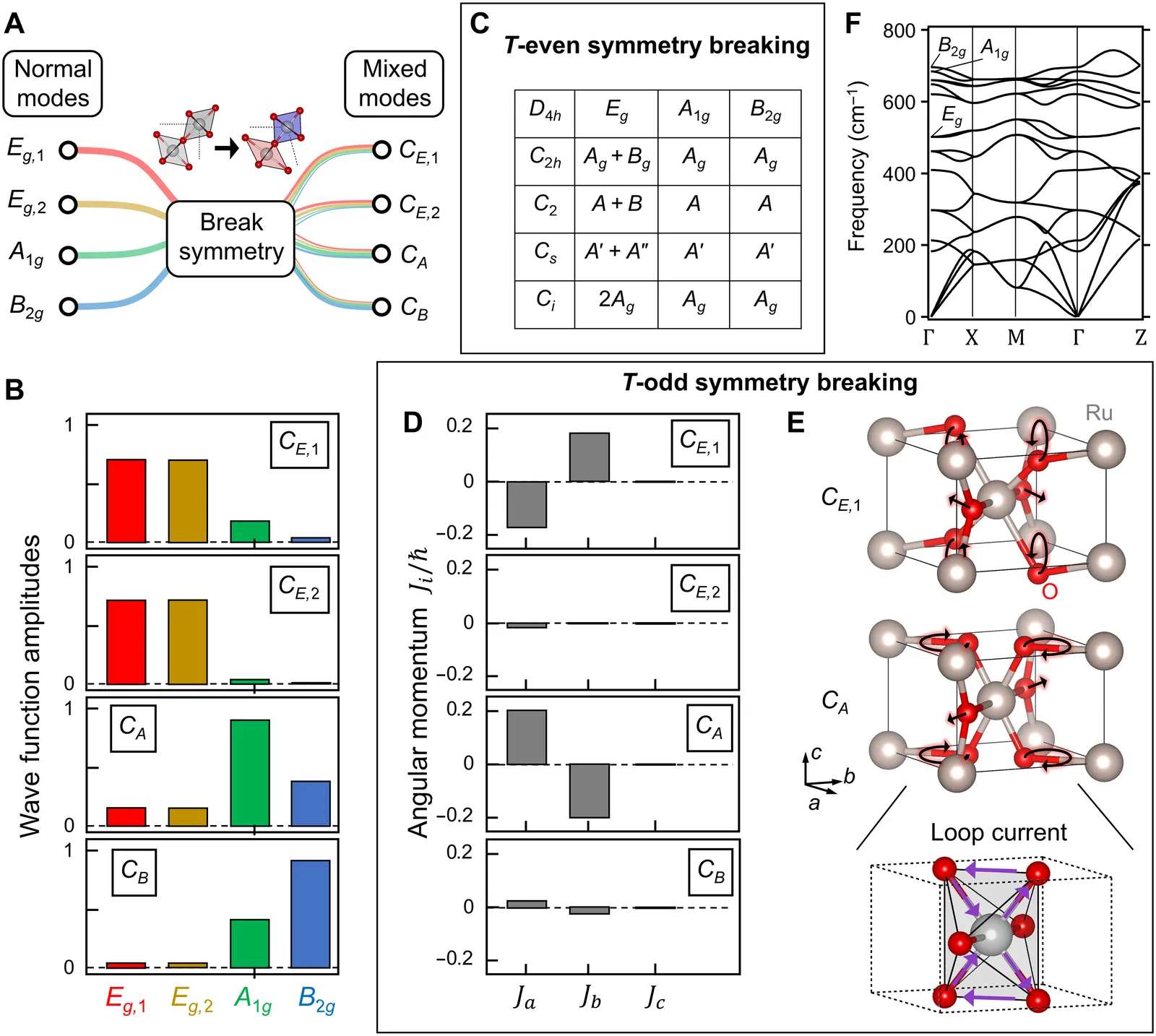
Possible symmetry-breaking mechanisms responsible for the selection rule breakdown and ROA. (**A**) Phonon normal modes derived from the crystallographic point group (left) undergo coherent mixing due to symmetry breaking, yielding hybridized modes (right). (**B**) Weights of the four basis modes in each hybridized mode. (**C**) T-even symmetry-breaking mechanism: possible subgroups of $D_{4h}$ (excluding the trivial $C_{1}$ case) and a correlation table showing how irreducible representations descend into each subgroup. (**D** and **E**) T-odd symmetry-breaking mechanism. (D) Angular momenta of the hybridized phonon modes, projected onto the crystallographic *a*, *b*, and *c* axes. (**E**) Atomic trajectories for the $C_{E,1}$ and $C_{A}$ modes, with an inset showing an example of an orbital loop current pattern that breaks T, analogous to the LC-$\Theta_{\text{II}}$ phase proposed for cuprates (*69*). (**F**) Phonon dispersion relation computed by DFPT for the fully relaxed undistorted $D_{4h}$ structure. The absence of branches with negative frequencies or soft modes suggests no structural instability of purely lattice origin. (B) to (E) are determined from best fits to experimental data.

The symmetry breaking event central to this interpretation can arise from two broad classes: a T-even mechanism, which purely breaks spatial symmetry, or a T-odd mechanism, which violates time-reversal symmetry in a manner analogous to magnetism.

Within the T-even symmetry breaking mechanism, we consider coherent coupling between the phonon normal modes, described by the eigenvalue equation

$${\text{Du}}_{m}=\omega_{m}^{2}u_{m}$$ (5)

which yields the eigenvectors **u***_m_* for the hybridized modes ($m=C_{E,1},C_{E,2},C_{A},C_{B}$). Here, the dynamical matrix is given by

$$D=\left(\begin{array}{cccc} \omega_{A_{1g}}^{2} & g_{\mathrm{AB}} & g_{\mathrm{AE}} & g_{\mathrm{AE}}\\ g_{\mathrm{AB}} & \omega_{B_{2g}}^{2} & g_{\mathrm{BE}} & g_{\mathrm{BE}}\\ g_{\mathrm{AE}} & g_{\mathrm{BE}} & \omega_{E_{g}}^{2} & g_{\mathrm{EE}}\\ g_{\mathrm{AE}} & g_{\mathrm{BE}} & g_{\mathrm{EE}} & \omega_{E_{g}}^{2} \end{array}\right)$$ (6)

where the diagonal entries are the squared natural frequencies of the uncoupled $D_{4h}$ modes and the off-diagonal elements $g_{\mathrm{AB}}$, $g_{\mathrm{AE}}$, $g_{\mathrm{BE}}$, and $g_{\mathrm{EE}}$ are coupling coefficients arising from the broken spatial symmetry. These coupling coefficients are fully determined by fitting to the experimental Raman data. From the resulting eigenvectors **u***_m_*, we obtain the Raman tensors of the hybridized modes as linear combinations of the original tensors: ${\tilde{R}}_{m}=\sum_{n}u_{m,n}R_{n}$, where the index *n* runs over the original modes ($E_{g,1}$, $E_{g,2}$, $A_{1g}$, and $B_{2g}$). The magnitudes $|u_{m,n}|$ of these coefficients, representing the weight of each original mode in the new hybridized eigenvectors, are shown in Fig. 5B and reveal considerable mode mixing. From a group theory perspective, the new point group describing the spatial symmetry of RuO_2_ that enables these normal-mode couplings must be a subgroup of $D_{4h}$ in which the direct products between coupled modes contain the fully symmetric irreducible representation (*53*). All possible subgroups (excluding the trivial *C*_1_ case) satisfying this requirement are listed in Fig. 5C, together with a correlation table showing how the irreducible representations descend into each subgroup.

An alternative T-odd symmetry breaking mechanism could equivalently account for our data. Within the Born-Oppenheimer approximation for phonon excitations, the nuclear coordinates determine the instantaneous electronic ground state (*54*, *55*). In crystals with broken T, the electronic state back-acts on the nuclear dynamics by introducing a vector potential. This electron-nuclear back-action modifies the lattice equation of motion to

$${\dot{v}}_{i}^{\alpha }+K_{\mathrm{ij}}^{\alpha \beta }u_{j}^{\beta }+2{\tilde{A}}_{\mathrm{ij}}^{\alpha \beta }v_{j}^{\beta }=0$$ (7)

where *u* and *v* denote the nuclear positions and velocities, *i* and *j* are atomic indices, and α,β=x,y,z indicate directions. The first two terms are sufficient to describe conventional phonon dynamics, with $K_{\mathrm{ij}}^{\alpha \beta }$ being the force-constant matrix that characterizes the restoring forces. The third term, however, is nontrivial, as it describes the force on the *i*th atom along direction α due to the *j*th atom’s velocity along β. This term, representing the velocity-force coupling mechanism (*56*, *57*), is characterized by the antisymmetric matrix ${\tilde{A}}_{\mathrm{ij}}^{\alpha \beta }$ (with ${\tilde{A}}^{T}=-\tilde{A}$), which is only nonzero if T is broken and is proportional to the nuclear Berry curvature, $G_{\mathrm{ij}}^{\alpha \beta }=2\hbar \text{Im}\langle \partial_{\mathrm{i\alpha}}\psi \left(\vec{R}\right)|\partial_{\mathrm{j\beta}}\psi \left(\vec{R}\right)\rangle$, where $\psi \left(\vec{R}\right)$ is the electronic wave function parameterized by nuclear coordinates $\vec{R}$. Semiclassically, this velocity-force coupling acts as an effective magnetic field on each ion, distorting its otherwise linear motion into an elliptical/circular trajectory. Phonon angular momenta thereby emerge, giving rise to chiral phonon modes (*58*, *59*).

To present a phenomenological description to our observation, we developed a theory inspired by the phonon Hall effect (*60*, *61*). As detailed in the Supplementary Materials, this reduces to solving the eigenvalue equation

$$i\left(\begin{array}{cc} 0 & I\\ -D & -2A \end{array}\right)\left(\begin{array}{c} u_{m}\\ v_{m} \end{array}\right)=\omega \left(\begin{array}{c} u_{m}\\ v_{m} \end{array}\right)$$ (8)

where *I* is the identity matrix and **u***_m_* and **v***_m_* are the phonon displacement and velocity eigenvectors, respectively. D=diag $\left(\omega_{E_{g}}^{2},\right.\left.\omega_{E_{g}}^{2},\omega_{A_{1g}}^{2},\omega_{B_{2g}}^{2}\right)$is a diagonal matrix. The antisymmetric matrix *A*, derived from $\tilde{A}$ through linear transformations, encodes the velocity-force coupling. By treating the elements of *A* as fitting parameters, we recover mode-mixing physics analogous to the model in Eq. 5, giving fits to experimental polar plots with the same quality as in Fig. 2 (I to K). The resulting magnitudes |**u***_m_*| again give the weights of the original modes in the hybridized modes, yielding results identical to Fig. 5B. However, the phase factors of the complex-valued **u***_m_* differ between the T-odd and T-even cases. This distinction reflects the fact that time-reversal breaking induces not only phonon mixing but also phonon angular momenta, as shown in the calculations of Fig. 5D and the reconstructed atomic trajectories in Fig. 5E. Notably, the $C_{E,1}$ and $C_{A}$ modes have larger angular momenta than the others, and their atomic trajectories feature elliptical motion of O atoms about an axis lying in the *ab* plane. This arises because both modes originate from $E_{g}-A_{1g}$ mixing, where the linear motion of O atoms along *c* (*E_g_*) superposes with their in-plane motion (*A*_1*g*_) with a phase delay.

We now discuss the possible physical origins of the conjectured symmetry-breaking mechanisms. For the T-even case, although the data require a reduction of the crystallographic symmetry to some subgroup of $D_{4h}$, we do not attribute this to a structural distortion of the atomic lattice. Our powder x-ray (Fig. 1B) and single-crystal Laue diffraction (fig. S1) data both indicate a close match to the $D_{4h}$ point group. Furthermore, DFPT calculations performed on a fully relaxed $D_{4h}$ structure yield fully stable phonon dispersions with no imaginary frequencies or soft modes (Fig. 5F), ruling out lattice instability as the driving mechanism for our observed phenomena.

We next discuss the possibility of surface-related effects. A low-symmetry surface can host phenomena such as reconstruction, enhanced magnetism, or spin textures distinct from the bulk and could in principle relax Raman selection rules. For the purely structural scenario, we performed first-principles calculations of surface relaxation for both Ru-rich and O-rich terminations of the (111) surface (Supplementary Materials). Our results show that the surface lattice distortion decays sharply with depth, penetrating only a few Å into the crystal—orders of magnitude smaller than the optical penetration depth of our probe laser, which we estimate to be ∼58 nm (Supplementary Materials). This renders a purely structural surface origin unlikely. Turning to magnetism, bulk altermagnetism is ruled out by our photoemission characterization on the same growth batch of samples used in this study, which detects no spin splitting of bulk bands ; the same study shows that while the surface states exhibit spin polarization patterns, their momentum-dependent textures are fully consistent with a nonmagnetic surface description combined with Rashba spin-orbit coupling. Together, we tend to believe that surface effects—whether structural or magnetic in origin—are unlikely to account for our experimental data, although our measurements cannot definitively exclude them, particularly if they have exceptionally strong optical responses beyond what our current models capture. Given the ongoing discussion of novel magnetism on the RuO_2_ (110) surface (*62*, *63*), systematic measurements on (111)-cut thin-film samples of varying thicknesses would provide decisive information to determine the role of surface effects.

One bulk-dominated symmetry-breaking mechanism, applicable to both T-even and T-odd scenarios, is hidden order arising from multipolar moments of Ru^4+^. Previous studies have largely focused on local magnetic dipole moments, reflecting the community’s longstanding interest in long-range magnetic order. However, multipoles—known to underlie various forms of hidden order—may be critical here (*64*–*66*). Given the local site symmetry and crystal field environment of Ru^4+^, finite multipole moments of various orders must exist. Quadrupole moments, for instance, have been identified as essential in resonant x-ray scattering studies (*67*), and their ordering could serve as a T-even mechanism for spatial symmetry breaking. Octupole components, by contrast, can break T. Although ordering of certain octupole components has been associated with altermagnetism (*68*) and thus can be ruled out in our case, other irreducible components of the octupole tensor could potentially order. In addition, order parameters transforming as anapoles may arise from orbital loop currents circulating within the octahedra (Fig. 5E, inset), stabilizing toroidal moments (*69*–*71*). Precedent for such a loop-current phase exists in metallic ruthenates (*72*), and this picture can be consistent with existing neutron and μSR data, as these orders do not generate local moments or magnetic fields as strong as those produced by dipolar order. Previous studies on cuprates (*70*, *71*) have shown that multiple current patterns can yield distinct symmetry-breaking profiles, breaking not only T but also spatial-symmetry generators. Given that the observed stable phonon dispersion of RuO_2_ is inconsistent with lattice-driven instabilities, these possibilities, representing hidden symmetry breaking of electronic origin, warrant particular attention in future investigations. For instance, while our zone-center phonon measurements have not been able to differentiate the T-even and T-odd scenarios, our simplified model analysis suggests that finite-momentum phonon dispersion measurements—via inelastic neutron or x-ray scattering, for example—could in principle distinguish them (Supplementary Materials), as distinct phonon signatures are expected to emerge away from the zone center.

By performing RA-Raman measurements on zone-center phonons in RuO_2_, we uncovered anomalous angle-dependent scattering patterns and ROA that unambiguously signal a symmetry-breaking event overlooked in previous studies. Systematic analysis traces the origin to two broad classes of mechanisms—those that preserve time-reversal symmetry (T-even) and those that break it (T-odd). The former induces spatial symmetry reduction that descends the phonon irreducible representations to the same class, while the latter induces chiral phonons with finite angular momenta. Our findings point to two scenarios: Either surface-induced symmetry breaking produces an exceptionally strong optical response, or—more compellingly—hidden bulk orders, likely arising from multipolar moments, govern the observed behavior. These results establish RuO_2_ as a material where subtle symmetry breaking can manifest through lattice dynamics, eluding detection by conventional structural and magnetic probes. The presence of hidden order parameters may provide a unifying framework for interpreting a range of reported transport, spintronic, and optical phenomena in this compound. More broadly, our findings call for renewed experimental and theoretical efforts to map the phase diagram of RuO_2_ and related 4d transition metal oxides, where the interplay of spin-orbit coupling, crystal field effects, and electronic correlations may drive the system toward a variety of electronic instabilities, giving rise to unconventional phases of matter.

## MATERIALS AND METHODS

### Material growth and characterizations

Single crystals of RuO_2_ were synthesized by chemical vapor transport method. Polycrystalline RuO_2_ powder and TeCl_4_ powder were mixed and sealed into a silica tube under vacuum. After heating at 1050/950°C for 14 days, millimeter-size rod-like RuO_2_ single crystals were obtained. The orientations of the crystal axes were determined by Laue x-ray diffraction (fig. S1). The crystal was then cut along the [111] direction and polished to optical smoothness. The sample used for optical measurements had lateral dimensions of ∼2 mm by 2 mm. For temperature-dependent measurements, the crystal was mounted in a continuous-flow optical cryostat evacuated to a pressure better than 2 × 10^−6^ torr.

We performed temperature-dependent electrical transport measurements on the RuO_2_ single crystal. The resulting *R*-*T* curve, obtained via the standard four-probe method using a physical property measurement system, is presented in the inset to Fig. 1B. The measured residual resistivity ratio (*RRR* = 278) attests to the high quality of the crystals.

### Polarization configurations in RA-Raman measurements

The choice of polarization configuration in our RA-Raman spectrometer is crucial for determining which groups of Raman tensor elements are accessible in our measurements. As described in section “Sample and RA-Raman measurements,” the polarization states of both the incident laser and the analyzed scattered light can be selected from S, P, R, and L. Here, we clarify two points regarding the definition of these polarization configurations.

First, the linear polarizations S and P are uniquely defined relative to the scattering plane: S light has its electric field vector perpendicular to the scattering plane and parallel to the sample surface, while P light has its electric field vector parallel to the scattering plane and at an oblique angle to the sample surface. With this convention, the polarization states, when viewed from the laboratory frame, vary as the scattering-plane angle (φ) changes. Specifically, as φ sweeps from zero to 2π, the S and P polarizations trace out azimuthal and radial vector patterns, respectively.

Second, we clarify the conventions for defining the circular polarizations of light, which can depend on the choice of the frame of reference (FoR). In the FoR defined by light propagation, R light is defined, such that its electric field vector rotates in a right-handed sense relative to the direction of propagation; after reflection from a metallic mirror, R light becomes L light. In contrast, we adopt a fixed-source FoR, where the observer always looks along the optical axis toward the sample regardless of the light propagation direction. The RR configuration in our convention corresponds to the RL configuration in the FoR defined by light propagation. In our convention, the RL configuration specifically refers to cases where the helicity of the scattered light is reversed because of angular momentum transfer between photons and phonons, rather than as a result of mirror reflection. A total of eight polarization configurations can be defined: SS, SP, PS, PP, RR, RL, LR, and LL, where the first and second letters denote the incident and analyzer polarizations, respectively.

## Supplementary Materials

### This PDF file includes:

Supplementary Text Figs. S1 to S11 References
