# Supplementary material for: Anomalous phonon selection rules and Raman optical activity in RuO2
Source: Sci Adv. 2026 Oct 21;12(43):eaeh0887. doi: 10.1126/sciadv.aeh0887 (PMC13685927; doi:10.1126/sciadv.aeh0887)
Supplement: Supplementary file 1 [file sciadv.aeh0887_sm.pdf]

Science Advances
AAAS

Supplementary Materials for

Anomalous phonon selection rules and Raman optical activity in RuO_2

Di Cheng *et al.*

Corresponding author: Xinwei Li, xinweili@nus.edu.sg

Sci. Adv. **12**, eaeh0887 (2026)
DOI: 10.1126/sciadv.aeh0887

This PDF file includes:

Supplementary Text
Figs. S1 to S11
References

S1 Sample orientation by Laue x-ray diffraction

The Laue diffraction pattern identifies the in-plane projections of the RuO_2 crystal axes for the (111)-cut sample used in the RA-Raman measurements.

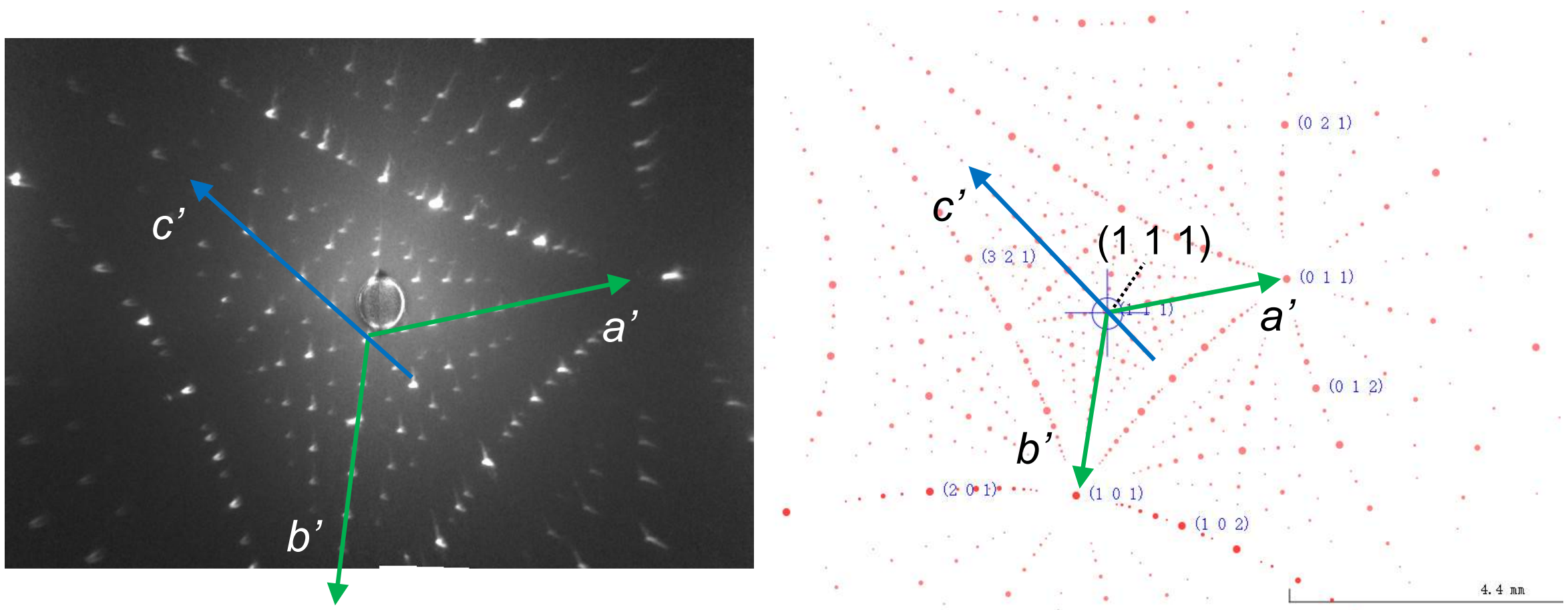

Figure S1: Laue pattern of a (111)-cut RuO_2 single crystal, marked with the directions of $\vec{a}'$, $\vec{b}'$, $\vec{c}'$, representing projections of the lattice vectors $\vec{a}$, $\vec{b}$, $\vec{c}$ onto the (111) plane.

S2 RA-Raman versus polarization-resolved Raman (P-Raman) measurements

The key distinction between conventional P-Raman spectroscopy and our RA-Raman measurements lies in the measurement geometry (*37*). In this section, we explain why RA-Raman offers significant advantages for resolving crystal symmetries.

Conventional P-Raman spectroscopy employs a normal-incidence geometry, where both the incident and detected scattered light are directed perpendicular to (from) the sample plane, restricting their electric fields to lie within the plane (*73*). In contrast, RA-Raman achieves well-controlled oblique incidence through a rotating optical assembly. There are two primary reasons why rotating the scattering plane under oblique incidence is superior to simply rotating the polarization under normal incidence.

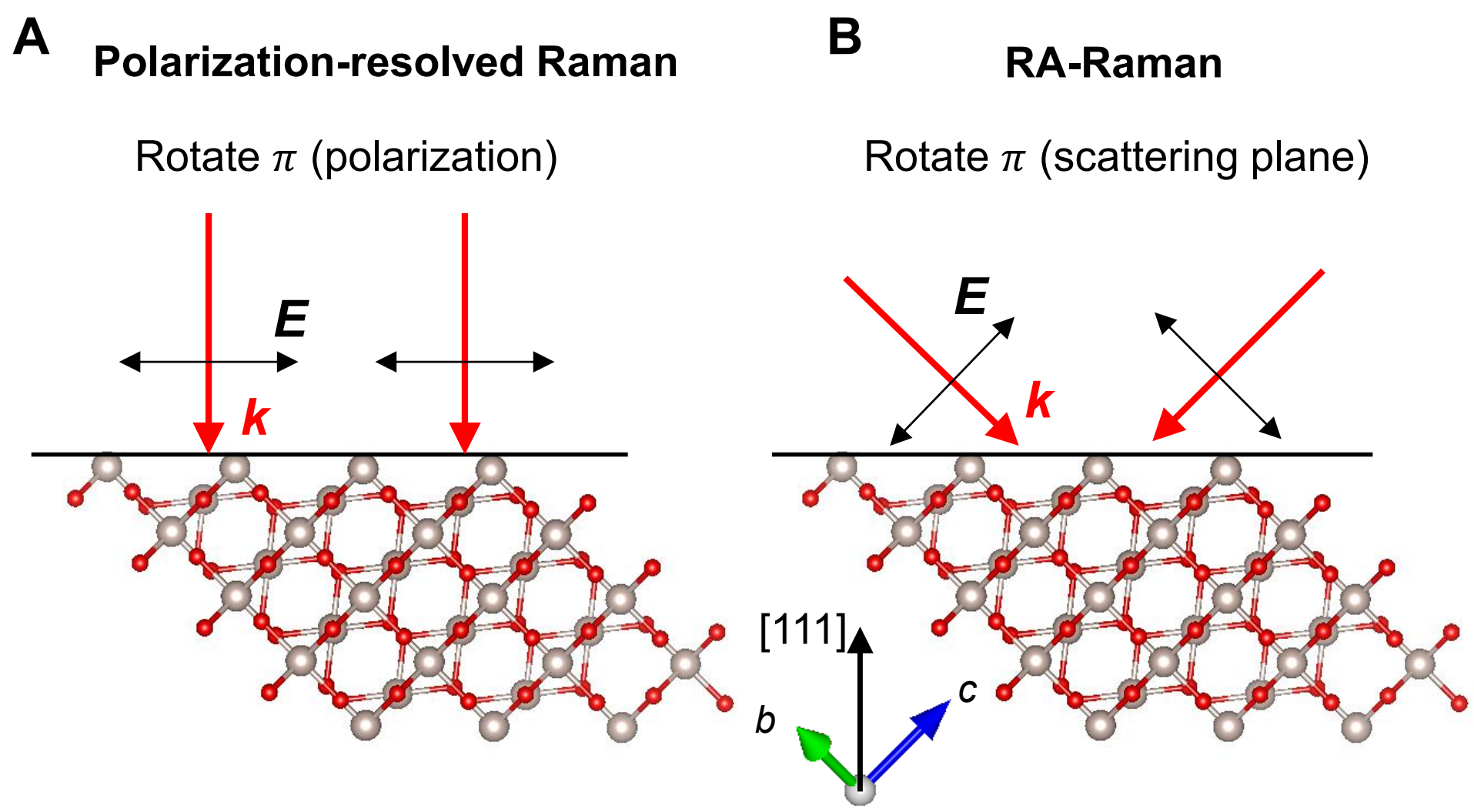

Figure S2: **Comparison of measurement geometries**. (**A**) Conventional P-Raman spectroscopy. (**B**) RA-Raman spectroscopy. Measurement configurations related by a π rotation of either the polarization (**A**) or the scattering plane (**B**) are shown side by side for direct comparison.

First, oblique incidence enhances accessibility to Raman tensor elements relevant to the scattering process. While the normal-incidence geometry can only couple to in-plane tensor elements (those with directional indices all in the sample plane), oblique incidence can couple to a broader set of tensor elements, including out-of-plane components.

Second, the inherent symmetry constraints of the two measurement geometries differ markedly. As illustrated in Fig. S2A, any normal-incidence optical measurement must exhibit C_2 symmetry, since the configuration repeats itself with every π rotation of the polarization. This limitation prevents such measurements from revealing C_3 symmetry (as in z-cut sapphire) or C_1 symmetry (as in (111)-cut RuO_2) even if these symmetries are present in the sample. The oblique-incidence geometry in RA-Raman measurements is not subject to this restriction. As shown in Fig. S2B, rotating the scattering plane by π results in distinct measurement configurations if the sample itself fails to preserve C_2; this can be visualized by considering the orientation of the propagation (k) and electric field (E) vectors relative to the crystal's short and long bonds. Consequently, the resolving power of RA-Raman measurements is far superior when investigating crystals with low symmetry, allowing unique in-plane axes and even specific atomic bond orientations to be readily identified by direct inspection of the polar plot patterns in various polarization configurations.

Finally, in RA-Raman measurements, we rotate the optical assembly rather than the sample to achieve scattering plane rotation. This approach, enabled by our alignment procedure, eliminates beam spot drift on the sample surface and ensures that the same region is consistently probed as the scattering plane is rotated (*74*).

The oblique incidence criterion $\theta \neq 0°$ is crucial for the RA-Raman instrument, as it is the key design feature that enables determination of the full 3×3 Raman tensor. At normal incidence ($\theta = 0°$), the geometry reduces to standard P-Raman, where only tensor elements within the two-dimensional polarization plane are probed.

To demonstrate this distinction, we performed measurements with RA-Raman at $\theta = 10°$ and P-Raman at $\theta = 0°$. The resulting intensity polar plots in the co-polarized P-in P-out geometry are summarized in Fig. S3 for the C_A (more A_{1g}-like) and C_B (more B_{2g}-like) modes. The $\theta = 10°$ data are nearly identical to those reported in the main text and are interpreted by our symmetry-breaking model, exhibiting a unique C_1 pattern (no rotational symmetry, only recovering after a full 360° rotation) symmetric about the unique in-plane $\vec{c}\,'$ axis. In contrast, the $\theta = 0°$ data recover the C_2 symmetry (recovering after 180° rotation), which is a hard requirement imposed by all P-Raman measurements because polarization is equivalent upon a π rotation. This C_2 symmetry loses information about the Raman tensor: for instance, the unequal lobes for the C_A mode in the $\theta = 10°$ case empirically convey the relative magnitudes between tensor elements, such as χ_{zz}/χ_{xx}, whereas the C_2 constraint at $\theta = 0°$ clearly cannot convey this information. Similar considerations apply to other modes. Although theoretically only a small θ is needed to break the C_2 constraint and access out-of-plane tensor elements, practically, the more oblique the RA-Raman geometry, the greater the resolving power on all tensor elements.

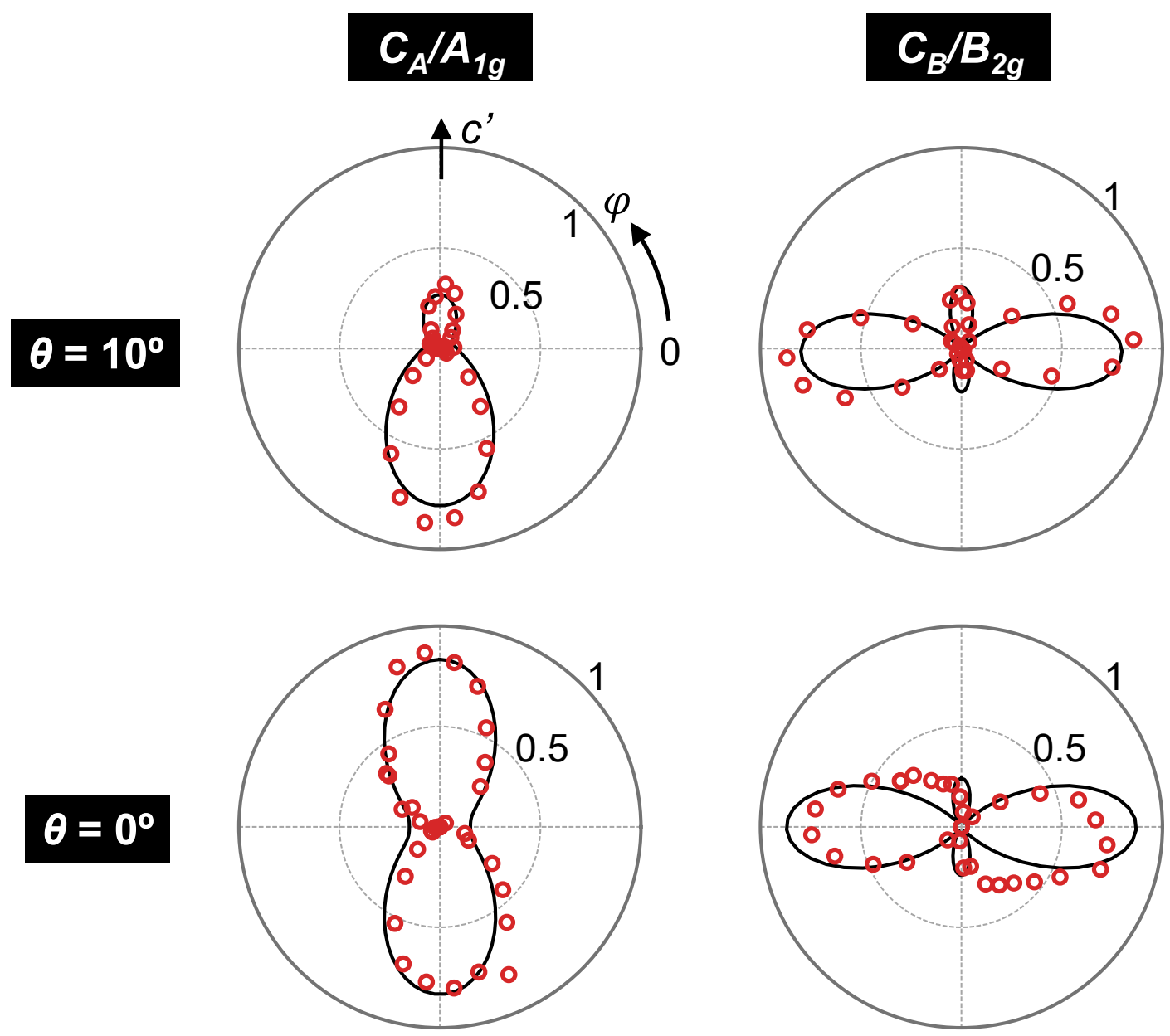

Figure S3: **Comparison of RA-Raman ($\theta = 10°$) and P-Raman ($\theta = 0°$) measurements.** Experimental polar plots (markers) and theoretical fits (lines) for the C_A and C_B modes at (top) $\theta = 10°$ (RA-Raman) and (bottom) $\theta = 0°$ (P-Raman). The $\theta = 10°$ data exhibit a C_1 pattern symmetric about the unique in-plane $\vec{c}'$ axis, whereas the $\theta = 0°$ data recover the C_2 symmetry imposed by the normal-incidence geometry.

S3 Ruling out the birefringence-induced Raman optical activity

The Raman optical activity (ROA), as defined in Eq. 2 of the main text, has traditionally played an important role in chirality-related physics and chemistry, yielding nonzero values for materials that are either magnetic (*41, 75*) or structurally chiral (*76*). In recent years, ROA is also recognized as a measure of Raman circular polarization (*77*), sensitively capturing $\mathcal{T}$-breaking responses in magnetic crystals. However, a notable caveat is that optical birefringence – when combined with oblique incidence – can produce nonzero ROA even in collective modes that are neither magnetic nor chiral (*46, 78*). Classically, this occurs due to polarization conversion effects arising in crystals with differing refractive indices for the ordinary and extraordinary rays. From a symmetry perspective, this can be attributed to the low rotational symmetry of the combined system (crystal plus measurement geometry), which removes the requirement for angular momentum conservation (*79*) (and thus L light can convert to R light, and vice versa). In this section, we address and rule out the possibility of birefringence-induced ROA in our measurements.

Previous studies have shown that for light propagating in uniaxial crystals (including RuO_2), polarization conversion primarily depends on the difference between ordinary and extraordinary refractive indices, as well as the propagation length (*46*). Specifically, the phase shift between ordinary and extraordinary rays serves as a key indicator:

$$\delta = k_0[n_o - n_e(\gamma)]s, \tag{S1}$$

where $k_0 = 2\pi/\lambda$ is the free-space wave-vector and s represents the propagation distance. n_o (n_e) is the refractive index for the ordinary (extraordinary) ray; n_e is a function of γ, the angle between the propagation vector and the optical axis (c-axis in RuO_2). For our experimental conditions, we find that the estimated δ is much less than one.

First, the upper bound for $n_o - n_e(\gamma)$ is given by the refractive index difference between $E \parallel a/b$ and $E \parallel c$. Optical data from both experimental broadband reflectivity measurements (*47, 80*) and first-principles density functional theory (DFT) calculations (*81*) indicate that RuO_2 exhibits nearly isotropic optical properties at our probe wavelength ($\lambda = 633$ nm), which falls within a crossover regime between intra-t_{2g} orbital transitions and the $t_{2g} \to e_g$ interband transitions. Consequently, $n_o - n_e \ll 1$.

Second, since RuO_2 is a metallic crystal, s should be given by the penetration depth of our probe

light. Adopting the optical constants from experimental data (*80*), we can perform an estimate:

$$s = \frac{1}{\mathrm{Im}(k_c)} = \frac{c}{\mathrm{Im}(n)\omega} \approx 58 \text{ nm}, \tag{S2}$$

where k_c is the photon wavevector in the crystal. Since $\lambda = 633$ nm for the probe wavelength, we can conclude that $k_0 s \ll 1$ in Eq. S1.

To ensure that polarization conversion is negligible, we performed rotational-anisotropy reflectivity (RA-reflectivity) measurements. The working principle of RA-reflectivity is similar to that of RA-Raman, except that we measure the power of the reflected light instead of the backscattered Raman signal, thereby obtaining the φ-dependent optical reflectivity $R(\varphi)$. As shown in Fig. S4, this measurement was carried out in four linearly polarized channels on our sample at room temperature. The key result is that the cross-polarized channels, $R_{\mathrm{SP}}(\varphi)$ and $R_{\mathrm{PS}}(\varphi)$, are at least two orders of magnitude weaker than the co-polarized channels, $R_{\mathrm{SS}}(\varphi)$ and $R_{\mathrm{PP}}(\varphi)$. This confirms that the polarization conversion between S and P light is negligible, a finding unexpected for a crystal with large birefringence at the probe wavelength. Additionally, $R_{\mathrm{SS}}(\varphi)$ and $R_{\mathrm{PP}}(\varphi)$ deviate only slightly from perfect circles; the ellipticity of these plots can be used to quantitatively estimate $n_o - n_e$. We present our method for performing this estimate below.

Following the derivation procedure in Ref. (*82*), the complex-valued reflection coefficients for a uniaxial crystal with arbitrary orientation are given by

$$r_{ss} = \frac{(q_1 - q_e)AE_y^e - (q_1 - q_o)BE_y^o}{D} \tag{S3}$$

$$r_{sp} = \frac{2n_1 k(AE_x^e - BE_x^o)}{D} \tag{S4}$$

$$r_{pp} = \frac{2q_1\left[(q_1 + q_e)E_x^o E_y^e - (q_1 + q_o)E_x^e E_y^o\right]}{D} - 1 \tag{S5}$$

$$r_{ps} = \frac{2n_1 k E_y^o E_y^e}{D}(q_e - q_o), \tag{S6}$$

where

$$\boldsymbol{E}^o = N_o \begin{pmatrix} -\beta q_o \\ \alpha q_o - \gamma K \\ \beta K \end{pmatrix} \tag{S7}$$

$$\boldsymbol{E}^e = N_e \begin{pmatrix} \alpha q_o^2 - \gamma q_e K \\ \beta \varepsilon_o k^2 \\ \gamma(\varepsilon_o k^2 - q_e^2) - \alpha q_e K \end{pmatrix} \tag{S8}$$

$$A = (q_o + q_1 + K \tan\theta) E_x^o - K E_z^o \tag{S9}$$

$$B = (q_e + q_1 + K \tan\theta) E_x^e - K E_z^e \tag{S10}$$

$$D = (q_1 + q_e) A E_y^e - (q_1 + q_o) B E_y^o \tag{S11}$$

$$K = k \sin\theta \tag{S12}$$

$$q_1 = k \cos\theta \tag{S13}$$

$$q_o^2 = \varepsilon_o k^2 - K^2 \tag{S14}$$

$$q_e^2 = q_o^2 + \frac{\Delta\varepsilon}{\varepsilon_o} \left[\varepsilon_o k^2 - (\gamma q_e + \alpha K)^2\right], \tag{S15}$$

and $\theta = 10°$ is the angle of incidence, k represent the photon wavevector in vacuum, ε_o and ε_e are, respectively, the dielectric constants for ordinary and extraordinary rays, $\Delta\varepsilon = \varepsilon_e - \varepsilon_o$, α, β, and γ are the directional cosines defining the crystal orientation, N_o and N_e are normalization factors. From these exact solutions we can obtain an approximate expression for r_{ss} as

$$r_{ss} \approx \frac{q_1 - q_o}{q_1 + q_o} \left(1 + \frac{\Delta\varepsilon}{\varepsilon_o} \frac{q_1}{q_o} \frac{\beta^2 \varepsilon_o}{\varepsilon_o - 1} \frac{\varepsilon_o k^2 - (\gamma q_o + \alpha K)^2}{\beta^2 \varepsilon_o k^2 + \alpha^2 q_o^2 - \gamma^2 K^2}\right). \tag{S16}$$

We can then invert this relation to express $\Delta\varepsilon$ versus r_{ss}:

$$\Delta\varepsilon = \frac{\varepsilon_o - 1}{\beta^2} \frac{q_o}{q_1} \frac{\beta^2 \varepsilon_o k^2 + \alpha^2 q_o^2 - \gamma^2 K^2}{\varepsilon_o k^2 - (\gamma q_o + \alpha K)^2} \left(\left|\frac{r_{ss1}}{r_{ss0}}\right| - 1\right)\Bigg|_{\varphi = \frac{\pi}{2}} \tag{S17}$$

where we have denoted $r_{ss0} = r_{ss}(\varphi = 0)$, $r_{ss1} = r_{ss}\left(\varphi = \frac{\pi}{2}\right)$, and assumed that r_{ss0} and r_{ss1} have similar phase. Substituting $\theta = 10°$, $r_{ss0} = 0.4e^{i\phi}$, and $r_{ss1}/r_{ss0} \approx \sqrt{1.13}$ (which is directly read from experimental data in Fig. S4), we numerically calculated $\frac{n_o}{n_e} = \frac{\Re(\sqrt{\varepsilon_o})}{\Re(\sqrt{\varepsilon_o + \Delta\varepsilon})}$. The result shows that $\left|\frac{n_o}{n_e} - 1\right| \leq 0.1$ under any ϕ. We numerically estimate $\left|\frac{n_o}{n_e} - 1\right| \approx 0.088 \ll 1$.

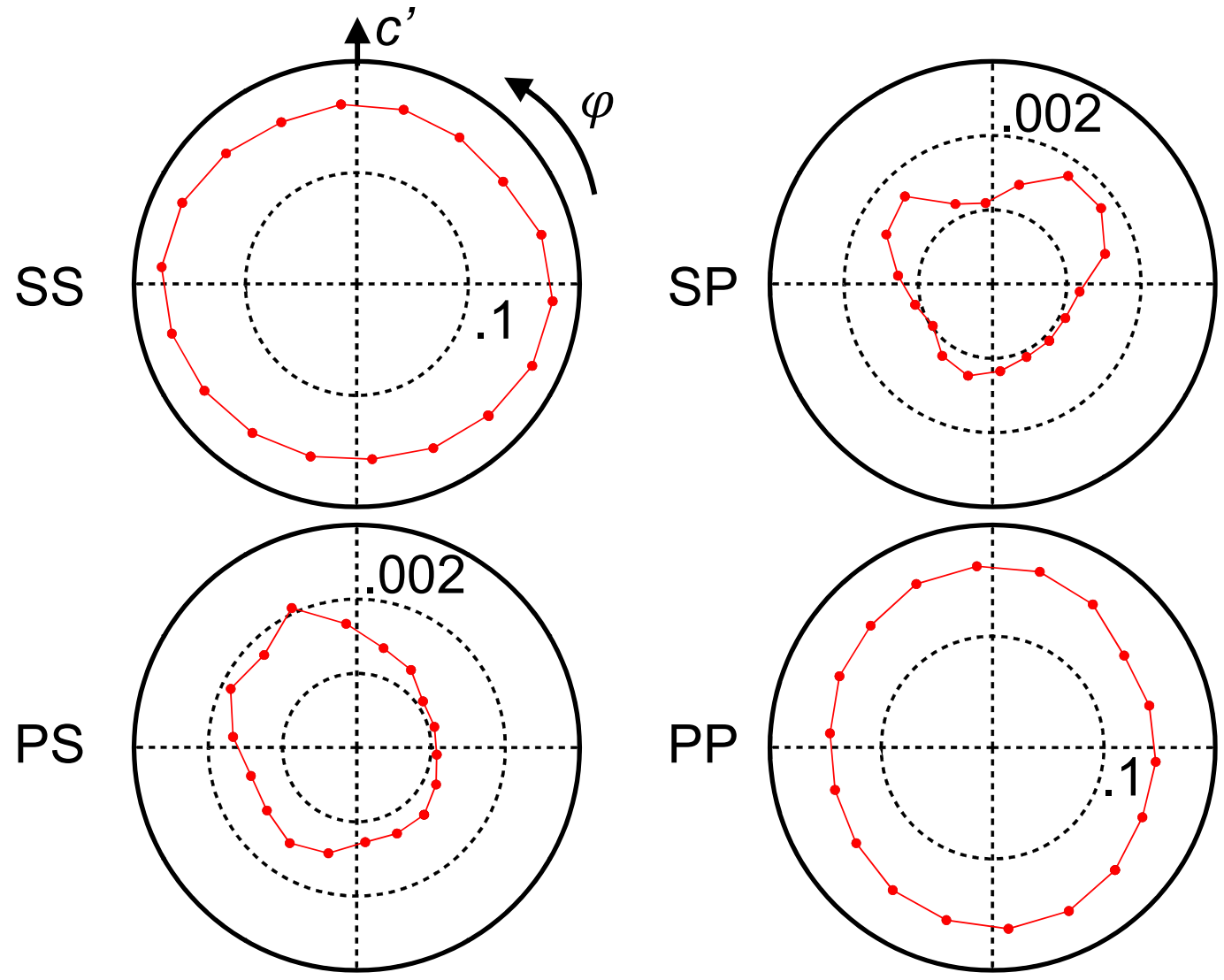

Figure S4: **φ-dependent RA-reflectivity polar plots for four linear polarization configurations**

S4 Calculation of Raman tensors from first principles

First-principles calculations were performed using DFT as implemented in the Vienna Ab initio Simulation Package (VASP) (*83, 84*) in spin-polarized version. The exchange-correlation interactions were treated with the generalized gradient approximation (GGA) in the Perdew-Burke-Ernzerhof (PBE) scheme (*85*). We incorporated spin-orbit coupling (SOC) and on-site Coulomb interactions through the DFT+U formalism (*86*), where an effective Hubbard parameter can be varied between U = 0 − 2 eV for the Ru 4d orbitals (*20, 31*). For small U values, the system would converge to a non-magnetic metallic ground state, which is assumed in this work.

The investigated RuO_2 adopts a rutile structure in a tetragonal unit cell in space group $P4_2/mnm$, with an optimized lattice of $a = b = 4.52$ Å and $c = 3.13$ Å. The structural optimization was performed using a plane-wave cutoff energy of 500 eV. The Brillouin zone was sampled with an $8 \times 8 \times 12$ Monkhorst-Pack k-point grid for the unit cell. To ensure a precise phonon ground state, the crystal structure was fully relaxed until the Hellmann-Feynman forces on each atom were less than 10^{-5} eV/Å. The electronic self-consistency cycle was considered converged when the total energy change fell below 10^{-8} eV.

Phonon frequencies and eigenvectors were computed using density functional perturbation theory (DFPT) (*87, 88*) as implemented in VASP. A Γ-only k-point grid was utilized for the

$4 \times 4 \times 4$ supercell to ensure well-converged phonon dispersions. By analyzing the symmetry of the calculated vibrational modes, we identified the irreducible representations (IRs) corresponding to the Raman-active modes. To simulate the Raman response, we performed frozen-phonon lattice modulations by displacing atoms along the normal coordinates of specific modes (*52, 89–93*). The displacement amplitudes were set to mimic a few-phonon excitation to remain within the harmonic regime. We verified that the potential energy surface for all investigated modes remained strictly parabolic under these modulations.

The optical properties were determined by calculating the frequency-dependent complex dielectric tensor $\varepsilon(\omega)$ using the Kubo-Greenwood formula (*94*). The imaginary part $\mathrm{Im}\,\varepsilon_{\alpha\beta}(\omega)$ was evaluated by a summation over transition matrix elements:

$$\mathrm{Im}\,\varepsilon_{\alpha\beta}(\omega) = \frac{4\pi^2 e^2}{\Omega m^2 \omega^2} \sum_{c,v,\mathbf{k}} \langle \psi_{c\mathbf{k}} | \hat{p}_\alpha | \psi_{v\mathbf{k}} \rangle \langle \psi_{v\mathbf{k}} | \hat{p}_\beta | \psi_{c\mathbf{k}} \rangle \delta(E_{c\mathbf{k}} - E_{v\mathbf{k}} - \hbar\omega) \tag{S18}$$

where Ω is the unit cell volume, $\psi_{c\mathbf{k}}$ and $\psi_{v\mathbf{k}}$ are the conduction and valence band wavefunctions, and $\hat{p}$ is the momentum operator. The real part of the dielectric tensor was subsequently obtained via the Kramers-Kronig (KK) relation (*94*):

$$\mathrm{Re}\,\varepsilon_{\alpha\beta}(\omega) = 1 + \frac{2}{\pi}\mathcal{P}\int_0^\infty \frac{\omega' \mathrm{Im}\,\varepsilon_{\alpha\beta}(\omega')}{\omega'^2 - \omega^2} d\omega' \tag{S19}$$

A dense k-point mesh of $12 \times 12 \times 16$ and 200 unoccupied bands were employed to ensure spectral convergence of the excited states.

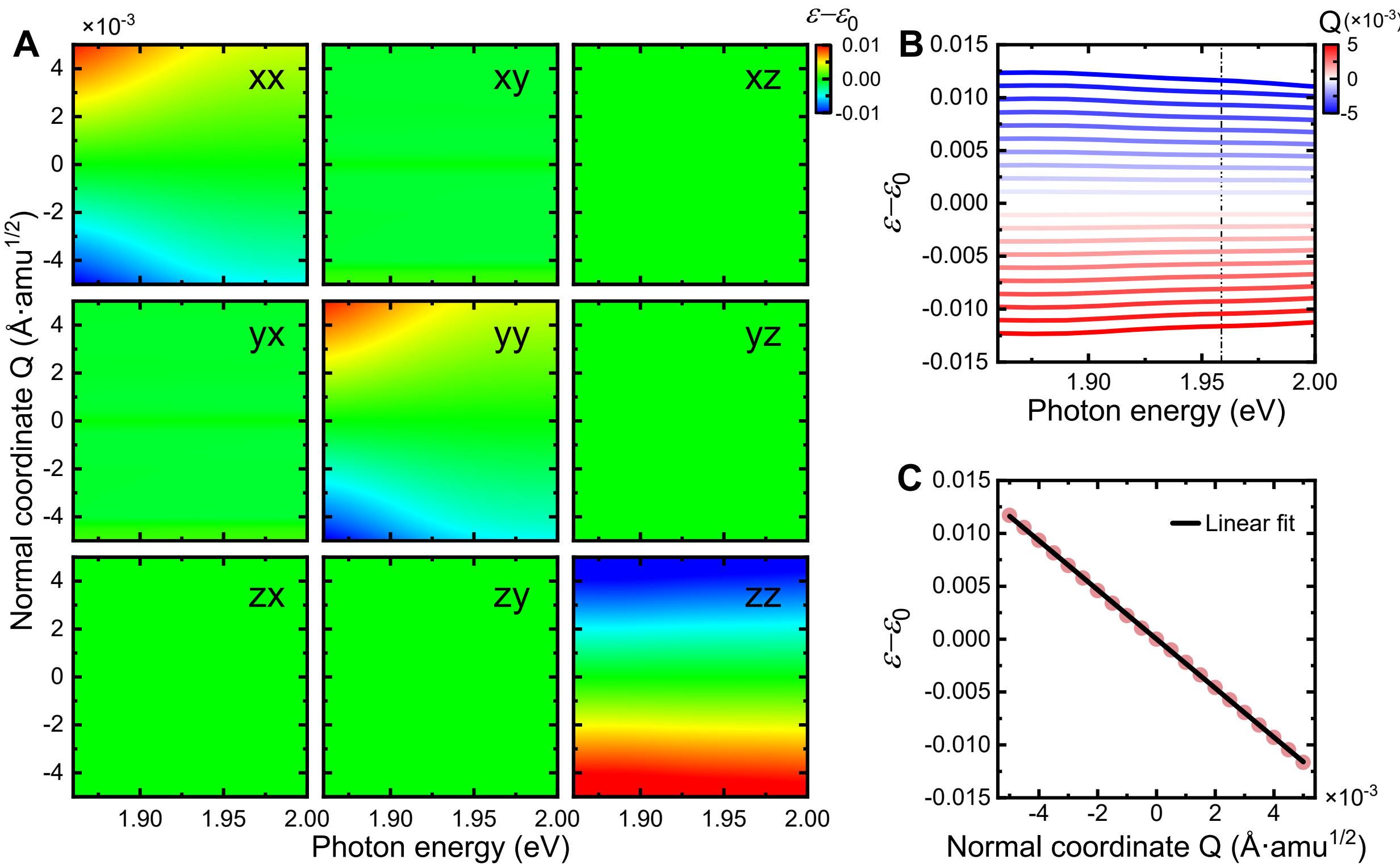

Figure S5: **Calculation of Raman tensors from first principles: an example of the A_{1g} mode.** (**A**) Evolution of the dielectric tensor as a function of photon energy (horizontal axis) and the collective coordinate of the A_{1g} phonon mode (vertical axis). The 3×3 grid corresponds to the nine components of the dielectric tensor. The colormap represents the interpolated difference in the dielectric response between the modulated structure and the equilibrium configuration. (**B**) Frequency-dependent variation of the zz component of the dielectric tensor, extracted from **A**, under various phonon displacements. The vertical gray line indicates the photon energy corresponding to a 633 nm incident laser. (**C**) Optical response at the 633 nm laser frequency as a function of the phonon displacement extracted from panel (B). The observed linear relationship between the dielectric tensor and the normal coordinate confirms that the slope corresponds to the Raman tensor element obtained from first-principles calculations. Normal coordinates are expressed in mass-reduced units, where amu stands for atomic mass unit (eg. 12 for carbon).

The Raman tensor components were extracted by computing the dielectric tensor $\varepsilon_{\alpha\beta}$ for a series of structures modulated by the normal coordinate Q, evaluated at a photon energy of 1.96 eV corresponding to the 633 nm laser excitation used in our experiments. An example of the calculated Q-dependence of the real part of $\varepsilon_{\alpha\beta}$ for the A_{1g} mode is shown in Fig. S5A. In the vicinity of the equilibrium position, the dielectric tensor elements exhibited a highly linear dependence on the modulation amplitude, as illustrated for representative zz component in Fig. S5B and C. The Raman tensor $\boldsymbol{R}$ for each mode was then determined from the slope of this linear relationship:

$$R_{\alpha\beta}(\omega) \propto \left.\frac{\partial \varepsilon_{\alpha\beta}(\omega, Q)}{\partial Q}\right|_{Q=0}, \tag{S20}$$

This approach enables the determination of complex-valued Raman tensors and facilitates direct comparison between the calculated tensor elements and the experimental results obtained from RA-Raman spectroscopy.

Another observation that can be made from Fig. S5 is that the Raman tensor structure remains robust across the entire wavelength range; although individual tensor elements show quantitative variation with photon energy, this affects only Raman intensity, not the selection rules. In addition, RuO_2 is a gapless metal with Dirac nodal lines near the Fermi surface. Optical excitations thus always involve a mixture of transitions across many bands, and the absence of an optical gap means that moderate wavelength tuning is unlikely to qualitatively alter this condition.

S5 Full data set interpreted by theory model

S5A Raman tensor theory

Raman spectroscopy measurements typically involve only very small momentum transfers. In centrosymmetric crystals such as RuO_2, these momentum transfers have negligible effects, as their influence on phonon polarization and frequency is rigorously proven to vanish to linear order (the leading order); see main text for a brief derivation. Thus, the probed phonon modes can be considered strictly at the zone center, where the Raman polarization selection rules are directly governed by Raman tensor theory (*1*). The model gives Raman scattering intensities

$$I_{E_g} \propto |\boldsymbol{e}_s^{\dagger} \cdot \boldsymbol{R}_{E_{g,1}} \cdot \boldsymbol{e}_i|^2 + |\boldsymbol{e}_s^{\dagger} \cdot \boldsymbol{R}_{E_{g,2}} \cdot \boldsymbol{e}_i|^2, \tag{S21}$$

$$I_{A_{1g}} \propto |\boldsymbol{e}_s^\dagger \cdot \boldsymbol{R}_{A_{1g}} \cdot \boldsymbol{e}_i|^2, \tag{S22}$$

$$I_{B_{2g}} \propto |\boldsymbol{e}_s^\dagger \cdot \boldsymbol{R}_{B_{2g}} \cdot \boldsymbol{e}_i|^2, \tag{S23}$$

for the E_g, A_{1g}, and B_{2g} modes, respectively, where $\boldsymbol{e}_i$ and $\boldsymbol{e}_s$ represent the crystal-cut-dependent and φ-dependent incident and scattered polarization vectors.

Within the standard theory, the Raman tensors for the three phonon modes are tabulated as

$$\boldsymbol{R}_{E_{g,1}} = \begin{pmatrix} 0 & 0 & 0 \\ 0 & 0 & e \\ 0 & e & 0 \end{pmatrix}, \quad \boldsymbol{R}_{E_{g,2}} = \begin{pmatrix} 0 & 0 & -e \\ 0 & 0 & 0 \\ -e & 0 & 0 \end{pmatrix}, \tag{S24}$$

$$\boldsymbol{R}_{A_{1g}} = \begin{pmatrix} a & 0 & 0 \\ 0 & a & 0 \\ 0 & 0 & b \end{pmatrix}, \tag{S25}$$

$$\boldsymbol{R}_{B_{2g}} = \begin{pmatrix} 0 & d & 0 \\ d & 0 & 0 \\ 0 & 0 & 0 \end{pmatrix}. \tag{S26}$$

Within the theoretical interpretation incorporating symmetry breaking, we need to apply the Raman tensors corresponding to the mixed phonon modes ($C_{E,1}$, $C_{E,2}$, C_A, C_B): $\boldsymbol{R}_{E_{g,1}}$ and $\boldsymbol{R}_{E_{g,2}}$ is replaced by $\boldsymbol{R}_{C_{E,1}}$ and $\boldsymbol{R}_{C_{E,2}}$, $\boldsymbol{R}_{A_{1g}}$ is replaced by $\boldsymbol{R}_{C_A}$, and $\boldsymbol{R}_{B_{2g}}$ is replaced by $\boldsymbol{R}_{C_B}$.

S5B Full data set with fitting

In this subsection, we present the complete set of φ-dependent Raman scattering intensity polar plots from RA-Raman measurements on RuO_2 at 153 K. Figures S6 and S7 display the results for all phonon modes in the four linear polarization channels – $I_{\mathrm{SS}}(\varphi)$, $I_{\mathrm{SP}}(\varphi)$, $I_{\mathrm{PS}}(\varphi)$, and $I_{\mathrm{PP}}(\varphi)$ – and the four circular polarization channels – $I_{\mathrm{RR}}(\varphi)$, $I_{\mathrm{RL}}(\varphi)$, $I_{\mathrm{LR}}(\varphi)$, and $I_{\mathrm{LL}}(\varphi)$ – respectively. Only half of this data set is shown in Fig. 2 of the main text. Here, the modes are labeled using mixed phonon notation, which is appropriate since the curves are fitted assuming symmetry breaking. In Fig. 2 of the main text, however, the conventional normal mode labels (E_g, A_{1g}, B_{2g}) are retained to facilitate comparison with the past literature. Note that C_E is majorly E_g in character, C_A is majorly A_{1g} in character, and C_B is majorly B_{2g} in character (see Fig. 5B of the main text).

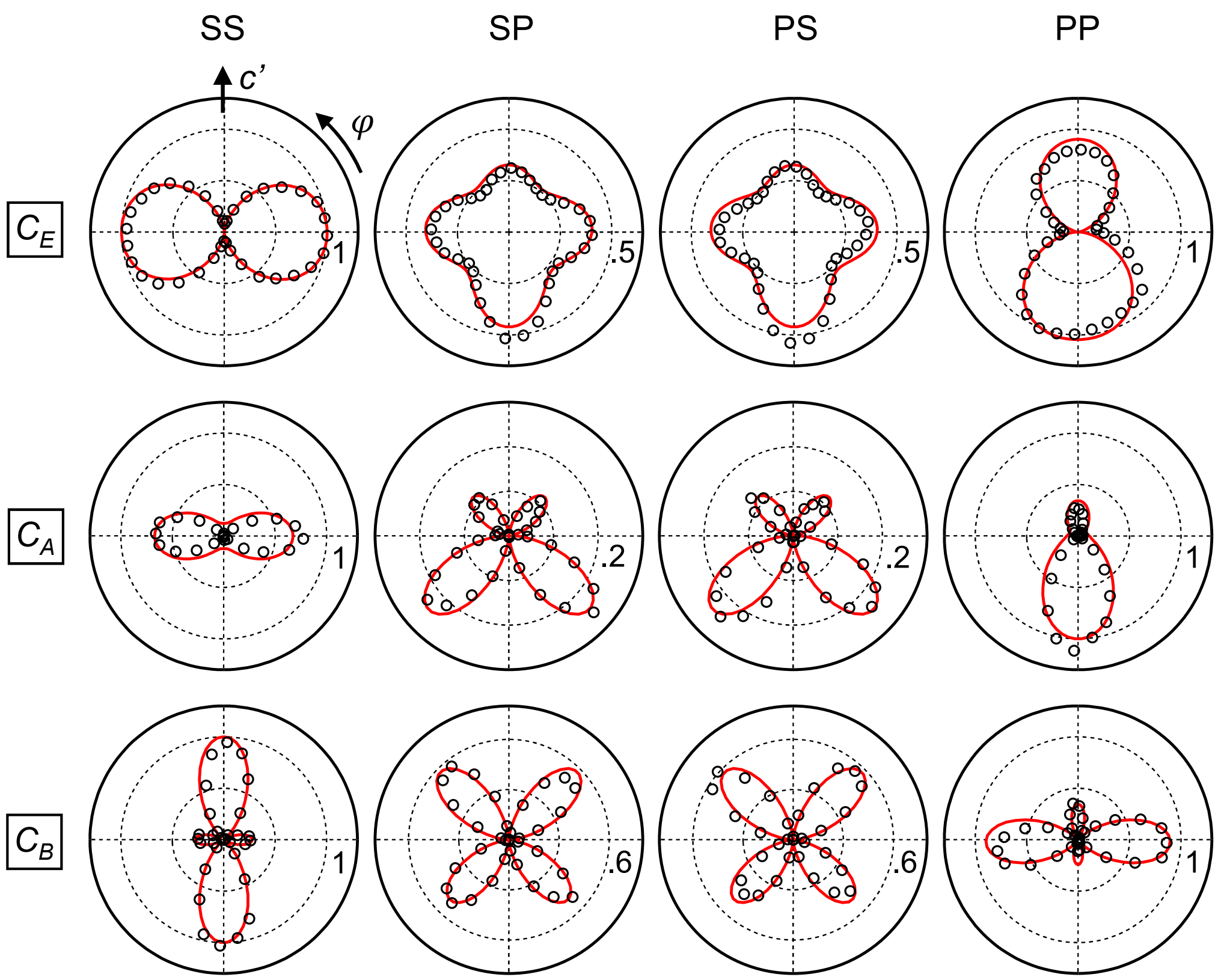

Figure S6: **φ-dependent scattering intensities for different phonon modes in four linearly polarized configurations.** Black circles: experimental data. Red lines: theoretical fits.

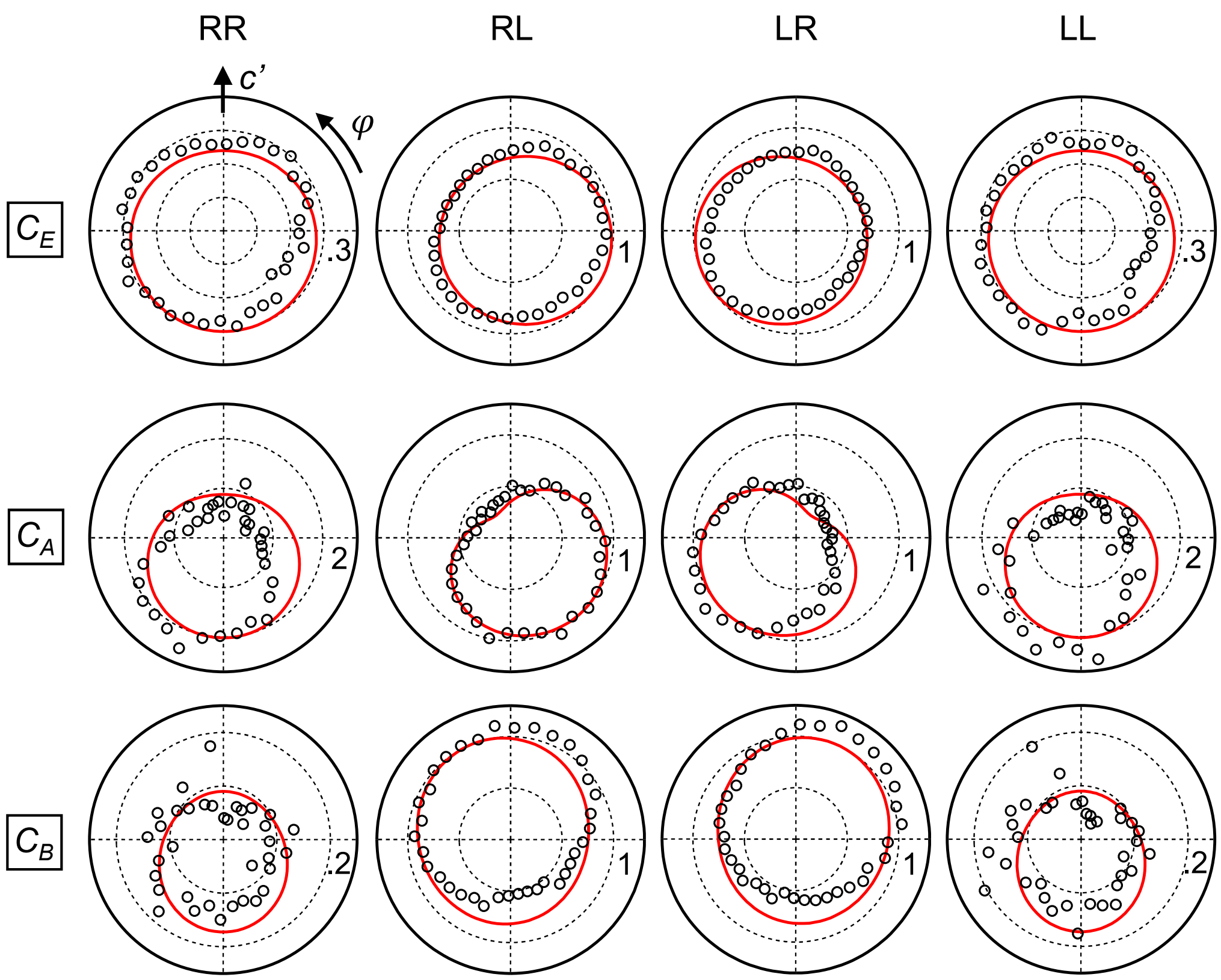

Figure S7: **φ-dependent scattering intensities for different phonon modes in four circularly polarized configurations.** Black circles: experimental data. Red lines: theoretical fits.

Theoretical fits to the polar plots are shown as red curves in Figs. S6 and S7. These fits are generated using either the $\mathcal{T}$-even spatial symmetry breaking model or the $\mathcal{T}$-odd model, both of which ultimately converge to identical optimal results. For each model, a single set of fitting parameters was used to achieve the best overall agreement with the entire dataset comprising 24 polar plots. The parameters include g_{EE}, g_{AE}, g_{BE}, and g_{AB} as introduced in Eq. 6 of the main text for the $\mathcal{T}$-even model, and the corresponding real-valued effective field parameters h_{EE}, h_{EA}, h_{EB}, and h_{AB} in Eq. S44 for the $\mathcal{T}$-odd model. Our fitting parameters also encompass the Raman tensor elements e, a, b, and d defined in Eqs. S24-S26. Flexibility in choosing the phase factors of the complex-valued Raman tensor elements allows the $\mathcal{T}$-even and $\mathcal{T}$-odd models to reach identical optimum fits, despite involving solutions to different eigenvalue equations.

S6 Effective Hamiltonian from phonon Hall effect theory

For the $\mathcal{T}$-odd model, the key phenomenology of mixed phonons in RuO_2 can be explained by the phonon Hall effect theory (*61, 95*), which describes the response of phonons to time-reversal symmetry breaking. The effect can be understood as arising from an effective internal magnetic field inherent to RuO_2; the problem shares the same effective Hamiltonian formalism (*96*). Here we present a brief recap of the procedure for deriving this effective Hamiltonian.

The starting point is a Hamiltonian describing the motion of charged ions in a magnetic field, which takes into account the kinetic and the potential energy terms

$$H = \frac{1}{2}(\boldsymbol{p} - \tilde{\boldsymbol{A}}\boldsymbol{u})^T(\boldsymbol{p} - \tilde{\boldsymbol{A}}\boldsymbol{u}) + \frac{1}{2}\boldsymbol{u}^T\boldsymbol{K}\boldsymbol{u} \tag{S27}$$

$$= \frac{1}{2}\boldsymbol{p}^T\boldsymbol{p} + \frac{1}{2}\boldsymbol{u}^T(\boldsymbol{K} - \tilde{\boldsymbol{A}}^2)\boldsymbol{u} + \boldsymbol{u}^T\tilde{\boldsymbol{A}}\boldsymbol{p}, \tag{S28}$$

where $\boldsymbol{u}$ is a column vector incorporating the mass-normalized displacements of all ions along all degrees of freedom, $\boldsymbol{p}$ is the corresponding conjugate momentum column vector, $\boldsymbol{K}$ is the real-space force constant matrix, and $\tilde{\boldsymbol{A}}$ is an antisymmetric real matrix which takes a block-diagonal form, with sub-matrices as elements

$$\Lambda_i = \begin{pmatrix} 0 & h_z^{(i)} & -h_y^{(i)} \\ -h_z^{(i)} & 0 & h_x^{(i)} \\ h_y^{(i)} & -h_x^{(i)} & 0 \end{pmatrix} \tag{S29}$$

arranged along the diagonal. The terms

$$h^{(i)}_{x,y,z} = -q_i B^{(i)}_{x,y,z}/(2m_i) \tag{S30}$$

accounts for the effective field $B^{(i)}_{x,y,z}$ experienced by the i-th ion, which is multiplied to the ion charge $-q_i$ and normalized by ion mass m_i to give the unit of frequency. The term $\boldsymbol{u}^T\tilde{\boldsymbol{A}}\boldsymbol{p}$ in Eq. S28 is referred to as the spin-phonon interaction term in the phonon Hall effect theory (*97*), and is also the key that gives rise to the $\mathcal{T}$-breaking phonon response in RuO_2 for our case.

Given the Hamiltonian, the equations of motion can be written as

$$\dot{\boldsymbol{p}} = -(\boldsymbol{K} - \tilde{\boldsymbol{A}}^2)\boldsymbol{u} - \tilde{\boldsymbol{A}}\boldsymbol{p} \tag{S31}$$

$$\dot{\boldsymbol{u}} = \boldsymbol{p} - \tilde{\boldsymbol{A}}\boldsymbol{u}. \tag{S32}$$

By constructing a column vector $\boldsymbol{v}$ denoting ion velocity, this can be rearranged into

$$\boldsymbol{v} = \dot{\boldsymbol{u}} \tag{S33}$$

$$\dot{\boldsymbol{v}} = -\boldsymbol{K}\boldsymbol{u} - 2\tilde{\boldsymbol{A}}\boldsymbol{v}. \tag{S34}$$

Equation S34 gives the equation of motion for the coordinates

$$\dot{\boldsymbol{v}} + \boldsymbol{K}\boldsymbol{u} + 2\tilde{\boldsymbol{A}}\boldsymbol{v} = 0, \tag{S35}$$

which well incorporates the velocity-force coupling term (the third term) and the restoring-force term (the second term), as expected.

Previous phonon Hall effective theory (*61, 95*) suggests that a good way to solve the dynamics given these equations of motion is to convert the question into an eigenvalue problem in the reciprocal space through a basis transformation, with an effective Hamiltonian

$$H_{\text{eff}} = i\begin{pmatrix} 0 & \boldsymbol{I} \\ -\boldsymbol{K}_q & -2\tilde{\boldsymbol{A}}_q \end{pmatrix} \tag{S36}$$

which gives the time-dependent evolution

$$i\frac{\partial}{\partial t}\psi_q = H_{\text{eff}}\psi_q, \tag{S37}$$

of the state vector $\psi_q = (\boldsymbol{u}_q, \boldsymbol{v}_q)^\dagger$. The Fourier components $\tilde{\boldsymbol{A}}_q$ and $\boldsymbol{K}_q$ are obtained from the real-space quantities $\tilde{\boldsymbol{A}}$, $\boldsymbol{K}$ through a transformation

$$\boldsymbol{X}_q = \sum_l \boldsymbol{X}_l e^{-iqR_l}, \tag{S38}$$

where l is the unit cell index. $\boldsymbol{K}_q$ is known as the dynamical matrix. Further, by assuming that $\boldsymbol{u}_q$ and $\boldsymbol{v}_q$ have the time-harmonic form ($e^{-i\omega t}$), Eq. S37 becomes an eigenvalue equation

$$H_{\text{eff}}\psi_q = \omega\psi_q, \tag{S39}$$

which gives the frequencies ω of the phonon branches.

Near the zone center, $q \approx 0$, we keep only one Fourier component of all vectors and matrices. Notably, since the ion motions across all unit cells are the same at $q = 0$, we use $\boldsymbol{u}$ and $\boldsymbol{v}$ to represent $\boldsymbol{u}_{q=0}$ and $\boldsymbol{v}_{q=0}$ since they are equivalent upon a normalization of $\psi_{q=0}$ anyway. Equation S39 expanded in the matrix form is now

$$i\begin{pmatrix} 0 & \boldsymbol{I} \\ -\boldsymbol{K}_0 & -2\tilde{\boldsymbol{A}}_0 \end{pmatrix}\begin{pmatrix} \boldsymbol{u} \\ \boldsymbol{v} \end{pmatrix} = \omega\begin{pmatrix} \boldsymbol{u} \\ \boldsymbol{v} \end{pmatrix}. \tag{S40}$$

We can pick phonon normal modes that diagonalizes the dynamical matrix as $\boldsymbol{K}_0\boldsymbol{U}_m = \boldsymbol{U}_m\omega^2$, where m, being the mode index, can take IR labels such as E_g, A_{1g}, B_{2g}. Different $\boldsymbol{U}_m$'s altogether define a unitary transformation $\boldsymbol{U}$ which makes matrix

$$\boldsymbol{D} = \boldsymbol{U}^T\boldsymbol{K}_0\boldsymbol{U} = \begin{pmatrix} \omega_1^2 & & \\ & \omega_2^2 & \\ & & \dots \end{pmatrix} \tag{S41}$$

diagonal. We can use a unitary transformation

$$\boldsymbol{\Gamma} = \begin{pmatrix} \boldsymbol{U} & \\ & \boldsymbol{U} \end{pmatrix} \tag{S42}$$

to transform both sides of the matrix equation Eq. S40, resulting in a new expression

$$i\begin{pmatrix} 0 & \boldsymbol{I} \\ -\boldsymbol{D} & -2\boldsymbol{A} \end{pmatrix}\begin{pmatrix} \boldsymbol{u}_m \\ \boldsymbol{v}_m \end{pmatrix} = \omega\begin{pmatrix} \boldsymbol{u}_m \\ \boldsymbol{v}_m \end{pmatrix}. \tag{S43}$$

where $\boldsymbol{A} \equiv \boldsymbol{U}^T\tilde{\boldsymbol{A}}_0\boldsymbol{U}$, and $\boldsymbol{u}_m \equiv \boldsymbol{U}^T\boldsymbol{u}$ and $\boldsymbol{v}_m \equiv \boldsymbol{U}^T\boldsymbol{v}$ are the normal mode displacement vector and velocity vector, respectively. The $\boldsymbol{A}$ matrix, defined in the space of normal modes, inherits its antisymmetric property $\boldsymbol{A}^T = -\boldsymbol{A}$ from the $\tilde{\boldsymbol{A}}$ matrix in the original Hamiltonian,

$$\boldsymbol{A} = \begin{pmatrix} 0 & h_{EE} & h_{EA} & h_{EB} \\ -h_{EE} & 0 & h_{EA} & h_{EB} \\ -h_{EA} & -h_{EA} & 0 & h_{AB} \\ -h_{EB} & -h_{EB} & -h_{AB} & 0 \end{pmatrix} \tag{S44}$$

where the matrix elements within $\boldsymbol{A}$ are real-valued (because the unitary matrix $\boldsymbol{U}$ is real for zone-center normal modes) and capture the effective magnetic fields experienced by one phonon normal mode due to the displacement or velocity of another phonon normal mode. h_{EE}, h_{EA}, h_{EB}, and h_{AB} represent the effective fields acting between $E_{g,1}$ and $E_{g,2}$, $E_{g,1/2}$ and A_{1g}, $E_{g,1/2}$ and B_{2g}, and A_{1g} and B_{2g} modes, respectively. These elements have different physical meaning from elements in $\tilde{\boldsymbol{A}}$, for which each off-diagonal element represents the effective magnetic field experienced by one ion either due to an external field or the internal effective field contributed by the nuclear Berry curvature contribution.

Solving the eigenvalue equation yields four eigenvectors $(\boldsymbol{u_m}, \boldsymbol{v_m})^{\mathrm{T}}$ associated with four positive eigenfrequency solutions ω, from which the Raman tensors of the hybridized modes can be reconstructed (*98*) via

$$\tilde{\boldsymbol{R}}_m = \sum^{n} u_{m,n} \boldsymbol{R}_n, \tag{S45}$$

where $\tilde{\boldsymbol{R}}_m$ and $\boldsymbol{R}_n$ represent the coupled and uncoupled modes, respectively. Here, only the position vector $\boldsymbol{u_m}$ within the state vector $(\boldsymbol{u_m}, \boldsymbol{v_m})^{\mathrm{T}}$ is used in the calculation of the Raman tensor because Raman tensor is a measure of how the optical susceptibility of a material depends on atomic positions instead of atomic velocity. The velocity term $\boldsymbol{v_m}$ could couple to the magnetic field component of light, but its contribution is expected to be small.

Furthermore, given the eigenvectors, the angular momentum of the coupled phonon mode with index m can be obtained as

$$J_m^{\alpha} = -i\hbar \psi_m^{\dagger} \epsilon^{\alpha} \psi_m \tag{S46}$$

where $\alpha = x, y, z$ is the directional index, $\psi_m = \boldsymbol{U}\boldsymbol{u_m}$ which converts the eigenvectors from the normal mode space back to the atomic space, and

$$\epsilon^x = \begin{pmatrix} 0 & 0 & 0 \\ 0 & 0 & 1 \\ 0 & -1 & 0 \end{pmatrix}, \epsilon^y = \begin{pmatrix} 0 & 0 & -1 \\ 0 & 0 & 0 \\ 1 & 0 & 0 \end{pmatrix}, \epsilon^z = \begin{pmatrix} 0 & 1 & 0 \\ -1 & 0 & 0 \\ 0 & 0 & 0 \end{pmatrix} \tag{S47}$$

are angular momentum operators defined with respect to the real-space atomic polarization (*99*). They need to direct product with an identity matrix I_4 (4 is the number of atoms within a unit cell) to form matrices with appropriate dimension to be used in Eq. S46.

S7 $Cd_2Re_2O_7$ control experiment

We performed a new symmetry-compatible control experiment on $Cd_2Re_2O_7$, a crystal that adopts a tetragonal D_4 structure at low temperature, with the same (111) cut, to confirm that the reported anomalous phonon selection rules are specific to RuO_2. $Cd_2Re_2O_7$ (CRO) is a semi-metallic $5d$ heavy-transition metal oxide with a well-established structural phase diagram (*44*): it adopts a cubic O_h structure above 200 K (Phase I), a tetragonal D_{2d} structure between 120 and 200 K (Phase II), and a tetragonal D_4 structure below 120 K (Phase III).

As shown in Fig. S8, our CRO crystal is (111)-cut. At 309 K in the cubic Phase I, the surface features $\vec{a}'$, $\vec{b}'$, and $\vec{c}'$ axes, the in-plane projections of the equivalent cubic $\vec{a}$, $\vec{b}$, and $\vec{c}$ axes, exhibiting three-fold rotational symmetry (Fig. S8A). At 88 K in Phase III, symmetry breaking renders one lattice constant slightly different from the other two, driven by distortions of Re and O octahedra, yielding a tetragonal D_4 structure with the (111) cut still well defined for the same sample (Fig. S8B). This low-temperature tetragonal phase thus serves as a control for our RuO_2 (111) study.

As shown in Fig. S8C, we focus on two modes among the multiple peaks appearing in the Raman spectra of CRO. In the cubic Phase I, a triply degenerate T_{2g} mode at 455 cm^{-1} and an A_{1g} mode at 485 cm^{-1} are observed (*100*). Our RA-Raman data across four polarization channels (PP, PS, SP, SS) all show excellent agreement with theoretical calculations using the standard Raman tensor theory without phonon hybridization (Fig. S8D): the T_{2g} mode exhibits three-fold symmetry inherited from the cubic (111) surface, while the A_{1g} mode shows isotropic co-polarized channels (PP and SS) and vanishing cross-polarized intensity (PS and SP), all in full agreement with the model. Upon cooling to the tetragonal Phase III at 88 K, the in-plane axis $\vec{c}'$ becomes inequivalent to $\vec{a}'$ and $\vec{b}'$. From IR decomposition, the T_{2g} mode splits into a singlet B_2 and a doubly degenerate E mode, while the A_{1g} mode becomes A_1, all of which are traceable in the Raman spectrum (Fig. S8E). The same RA-Raman measurement for the $B_2 + E$ (combined intensity) and A_1 modes recovers the anticipated symmetry breaking: $\vec{c}'$ becomes the unique axis along which the patterns become asymmetric. Most importantly, all these phonons exactly follow Raman tensor theory using the tensors applicable to the reported D_4 group, without any unanticipated anomaly (Fig. S8F). This set of data thus demonstrates that the failure of the standard Raman tensor model in RuO_2 has

a nontrivial origin, not accounted for by instrumental artifact or any artifact that could be associated with tetragonal crystal systems cut along (111).

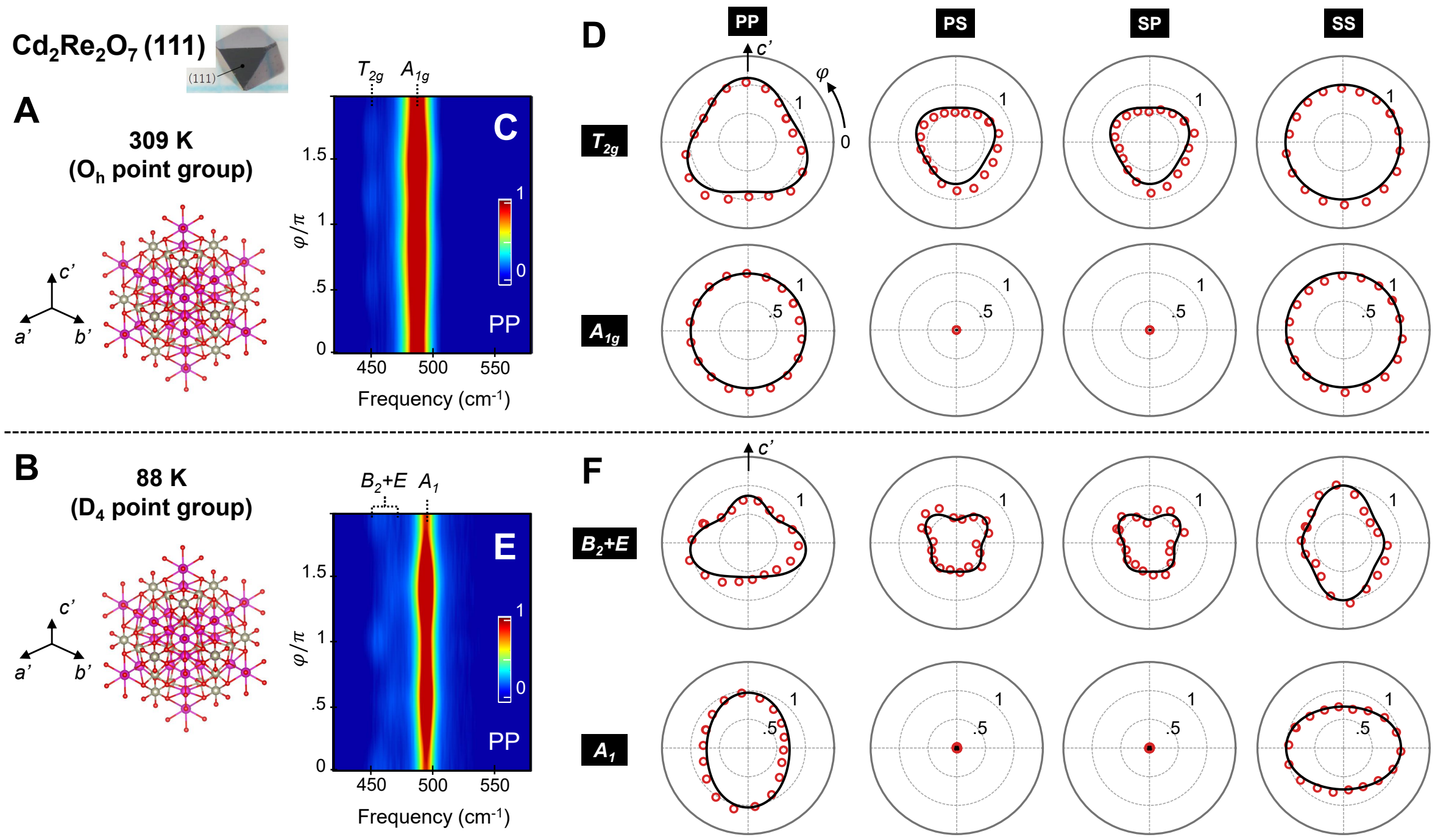

Figure S8: **Control experiment on (111)-cut $Cd_2Re_2O_7$.** (**A**) Cubic Phase I at 309 K (O_h point group), showing the in-plane projections $\vec{a}'$, $\vec{b}'$, $\vec{c}'$ of the cubic axes. (**B**) Tetragonal Phase III at 88 K (D_4 point group), showing the inequivalent $\vec{c}'$ axis. (**C**) φ-dependent Raman spectra (PP channel) in cubic Phase I, highlighting the T_{2g} and A_{1g} modes. (**D**) Experimental intensity polar plots (markers) and theoretical fits (lines) for the T_{2g} and A_{1g} modes in Phase I across four linear polarization channels (PP, PS, SP, SS). (**E**) φ-dependent Raman spectra (PP channel) in tetragonal Phase III, showing the decomposition of the T_{2g} mode into B_2 and E modes and the A_{1g} mode into A_1. (**F**) Experimental intensity polar plots (markers) and theoretical fits (lines) for the $B_2 + E$ and A_1 modes in Phase III.

S8 Surface reconstruction and optical probing depth

Clarifying the role of surface-related effects is crucial for identifying the origin of the symmetry breaking event leading to the anomalous phonon selection rules and ROA signal. While thickness-

dependent measurements on a series of thin films would be the most direct approach, such a study is beyond the scope of our current work due to the difficulty of thinning bulk RuO_2 single crystals to nanometer-scale thicknesses. Instead, we performed new first-principles calculations to quantify the expected extent of surface reconstruction.

As shown in Figs. S9A and B, the RuO_2 (111) surface can have two types of terminations: Ru-rich and O-rich. For both terminations, we constructed slab models stacking atomic layers (each 2.2 Å thick) along the surface normal beneath the surface, with a vacuum layer above. Along the lateral direction, a 2×2 supercell was used to accommodate potential surface reconstructions. First-principles DFT calculations were performed using the VASP package to examine native surface distortions. The exchange-correlation interactions were treated within the GGA using the PBE functional, and the core-valence interactions were described by the projector augmented-wave (PAW) method. A plane-wave cutoff energy of 500 eV was employed, and the Brillouin zone of the slab was sampled using a $3\times3\times1$ Γ-centered k-point mesh. During structural relaxation, the bottom layers were fixed to the bulk structure to simulate the pinning effect of the underlying substrate, while the top six atomic layers were fully relaxed until residual forces fell below 10^{-3} eV/Å.

Upon structural relaxation, Figs. S9C and D summarize the atomic displacements for Ru and O atoms relative to the bulk structure as a function of depth for both terminations. Although the top six layers were allowed to relax, significant atomic displacements only occur within the first three layers, merely 6.6 Å in both cases, and mostly stabilize to the bulk structure beyond. An exponential decay function fits the depth dependence well, confirming the highly localized nature of the surface reconstruction. Since the impacted thickness of ~6.6 Å is only about 1/100 of our optical penetration depth (~58 nm), it is unlikely that our signal is dominated by the surface reconstruction captured in our simulation.

We also offer some comments regarding the possibility of surface magnetism. Our unpublished spin-resolved angle-resolved photoemission spectroscopy (ARPES) characterization on the same growth batch of samples used in this study, which detects no spin splitting of bulk bands, shows that while the surface states exhibit spin polarization patterns, their momentum-dependent textures are fully consistent with a nonmagnetic surface description combined with Rashba SOC.

Altogether, we tend to believe that surface effects, whether structural or magnetic in origin, are unlikely to account for our experimental data. However, we acknowledge that our current

measurements cannot definitively exclude them, particularly if they possess exceptionally strong optical responses beyond what our current models capture. Systematic thin-film measurements on RuO_2 (111) with varying thicknesses would provide the definitive information.

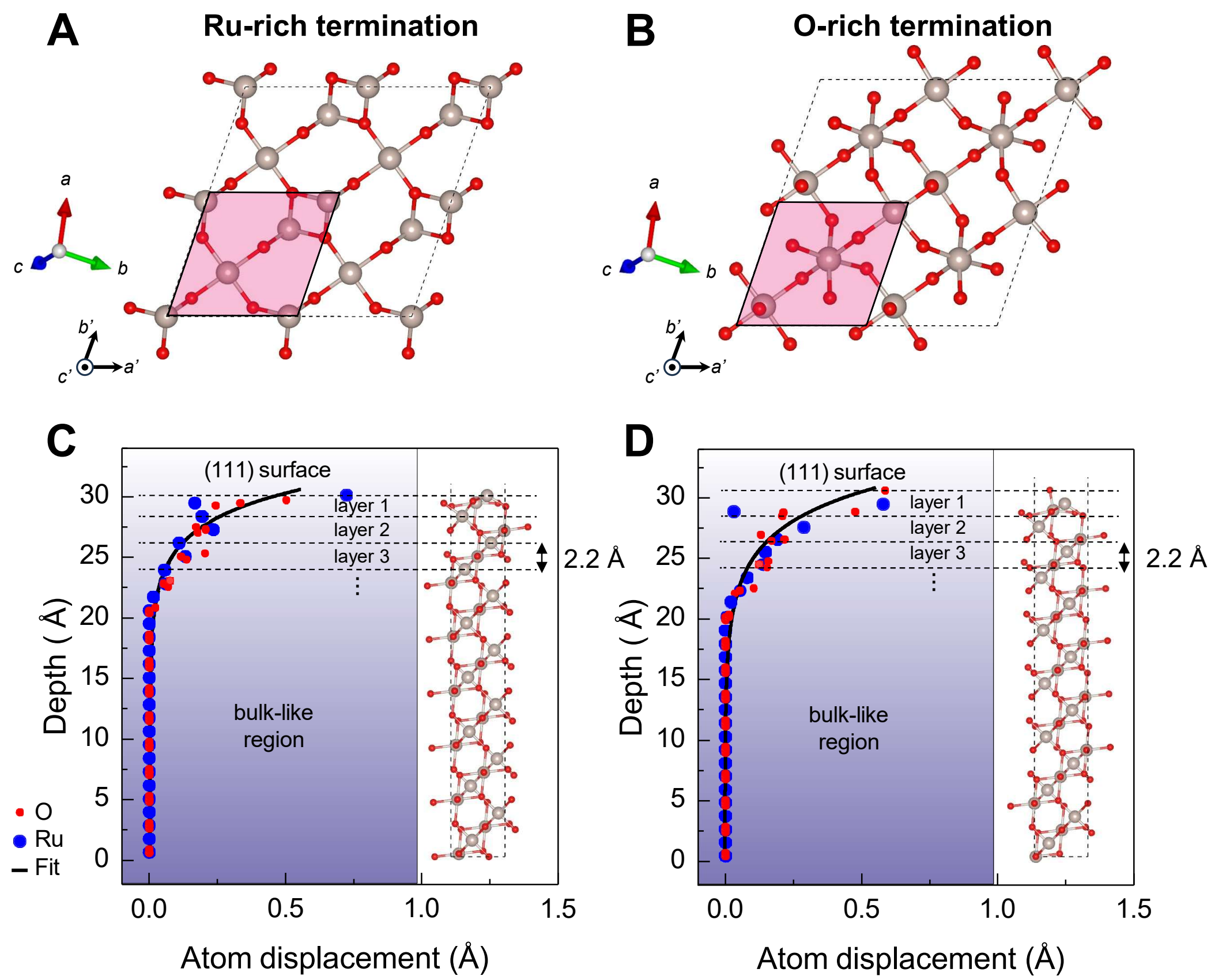

Figure S9: **First-principles calculations of surface reconstruction on RuO_2 (111).** Top-down view of the topmost atomic layer (2×2 supercell) for the (**A**) Ru-rich termination and (**B**) O-rich termination slab models. The $\vec{c}'$ axis is normal to the (111) plane. The red-shaded area marks the primitive unit cell, with $\vec{a}'$ and $\vec{b}'$ obtained from the transformation of the original lattice vectors $\vec{a}$, $\vec{b}$, $\vec{c}$. (**C**) Atomic displacements of Ru and O atoms relative to the bulk structure as a function of depth from the surface for the Ru-rich termination. (**D**) Same as (C) but for the O-rich termination. The solid lines represent exponential decay fits, confirming the highly localized nature of the surface reconstruction within the first three atomic layers (~6.6 Å).

S9 Phonon dispersion and lattice stability

To examine how the proposed symmetry breaking would modify the phonon dispersion away from the zone center, we performed DFT calculations for RuO_2 along a selected symmetry-breaking pathway for the time-reversal-even case. From the group-subgroup correlation table shown in Fig. 5C of the main text, we examined the $D_{4h} \to C_{2h}$ pathway, which from a symmetry perspective can explain the anomalous phonon mixing observed in our experiments. As shown in Fig. S10A, this descent requires an E_g distortion of the parent D_{4h} structure. Two pathways, $E_g(\eta, 0)$ and $E_g(\eta, \eta)$, can achieve this, corresponding to distortion along a single basis function or both components equally, yielding $C_{2h}(x)$ and $C_{2h}(xy)$, respectively, with the principal C_2 axis along the original in-plane lattice vector or the surface diagonal (Fig. S10B). Eigenmode analysis shows that the E_g distortion involves vertical displacements of O atoms (*101*). We applied a frozen phonon scheme to simulate how such distortion would impact the phonon dispersion. Note that here a pure structural distortion is simulated for ease of interpretation, while the actual time-reversal-even mechanism is likely driven by electronic correlations, an effect not captured by standard DFT treatments of RuO_2.

Figures S10C and D compare the phonon dispersion of the undistorted D_{4h} structure (black lines) with that of the distorted C_{2h} structures (red lines, with O displacements of 0.5% of the c-axis lattice constant) along the two E_g pathways. Figure S10E shows a standalone D_{4h} calculation with zone-center IRs labeled for reference. The computed results indicate that, beyond the expected zone-center degeneracy breaking, off-zone-center band modifications primarily affect the high-frequency phonon branches, altering band slopes and lifting degeneracies, with the most salient changes along the Γ–Z line, consistent with the vertical O displacements in the distorted structure.

For the time-reversal-odd case, we note that a full first-principles treatment of chiral phonons across the Brillouin zone would require calculating the antisymmetric matrix $\boldsymbol{A}_q$ (Eq. S36, with $\boldsymbol{A}_0$ already parameterized as fitting constants in our manuscript) from the nuclear Berry curvature, a calculation not yet widely implemented, though recent theoretical advancements have made significant progress in this direction (*57*). However, we expect the phonon dispersion in the time-reversal-odd scenario, manifesting as chiral phonon modes, to exhibit qualitative differences from the time-reversal-even mechanism described above. Off-zone-center measurements, potentially accessible via inelastic neutron or x-ray scattering, could thus serve as a distinguisher between the

two proposed mechanisms.

We also refer to the calculated phonon dispersion along the Γ-X-M-Γ-Z trajectory for the D_{4h} structure shown in Fig. S10E. The computation, performed using DFPT, was preceded by full structural relaxation to eliminate residual forces. A $3 \times 3 \times 3$ supercell was used to ensure force constants decay sufficiently to zero at the supercell boundaries, and the energy convergence criterion was set to 10^{-8} eV to guarantee accurate interatomic force constants. As shown in the figure, all phonon branches remain strictly positive throughout the entire Brillouin zone, and we observe no imaginary frequencies or soft modes. Our results, consistent with prior first-principles studies (*102–104*), demonstrate that the D_{4h} lattice is dynamically stable. This stability suggests that the symmetry breaking underlying our observed phonon mixing and ROA signal is unlikely to arise from a conventional lattice instability. Instead, a more subtle electronic or correlation-driven mechanism, such as hidden multipolar order, is the more plausible origin.

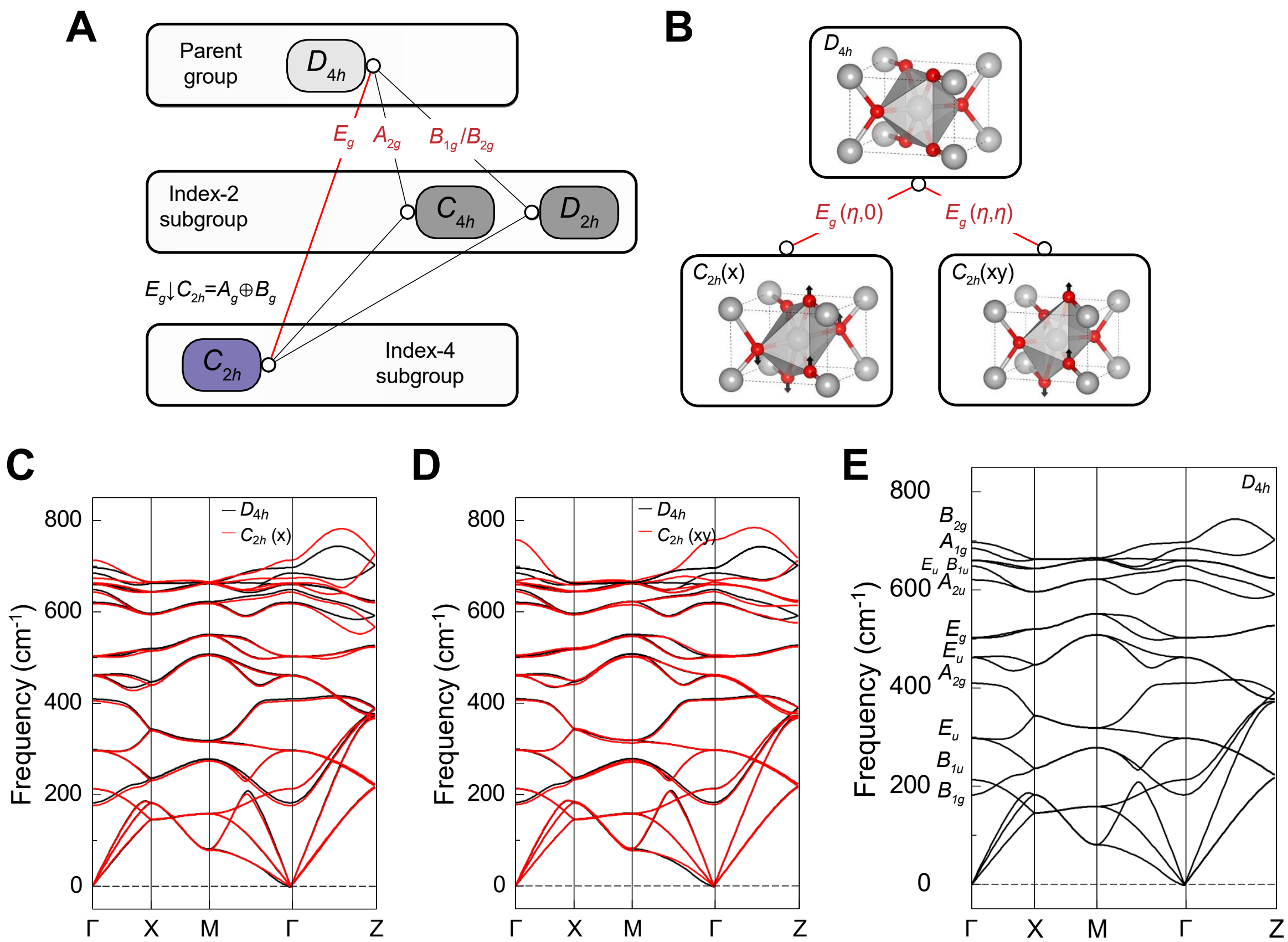

Figure S10: **Phonon dispersion calculations along the $D_{4h} \rightarrow C_{2h}$ symmetry-breaking pathway.** (**A**) Group-subgroup correlation diagram showing the $D_{4h} \rightarrow C_{2h}$ descent requiring an E_g distortion. (**B**) Schematic illustration of the two E_g distortion pathways from D_{4h}: $E_g(\eta, 0)$ and $E_g(\eta, \eta)$, yielding $C_{2h}(x)$ and $C_{2h}(xy)$, respectively. (**C**) Calculated phonon dispersion of the undistorted D_{4h} structure (black lines) compared with the distorted $C_{2h}(x)$ structure (red lines). (**D**) Same as (C) but for the distorted $C_{2h}(xy)$ structure. (**E**) Standalone D_{4h} phonon dispersion (same as black lines in C and D) with zone-center irreducible representations labeled for reference.

S10 Excitation-intensity-dependent RA-Raman data

Because RuO_2 is metallic, any photoinduced space-charge field would be efficiently screened by mobile carriers over the Thomas-Fermi screening length, which is much shorter than the optical penetration depth (~58 nm) of the laser. Its impact on the optical signal averaged over the probed depth would therefore be weak. We also performed an experimental test to rule out laser-induced effects.

As shown in Fig. S11, RA-Raman measurements were repeated at different excitation intensities, 100% power (same as the manuscript) and 50% power. The 50% power data were corrected for the longer integration time used in the measurement. The symmetry and shape of the scattering intensity polar plots remain unchanged, aside from an overall amplitude reduction proportional to the power decrease, confirming that the laser acts as a probe and does not actively alter the properties of the sample.

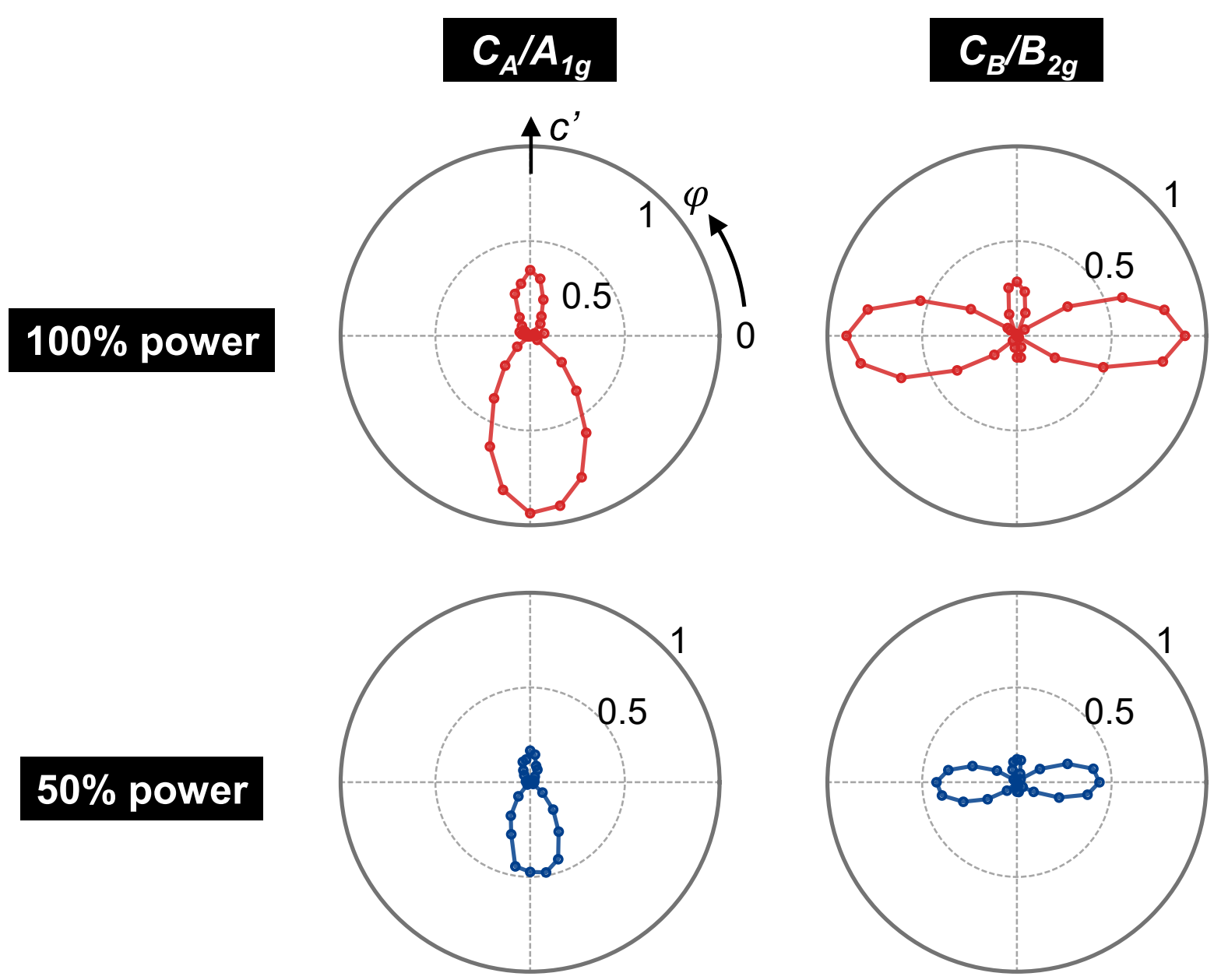

Figure S11: **Laser power dependence of RA-Raman measurements.** Experimental polar plots (PP channel) for the C_A and C_B modes at (top) 100% laser power (same as the main text) and (bottom) 50% laser power.

REFERENCES

1. R. Loudon, The Raman effect in crystals. *Adv. Phys.* **13**, 423–482 (1964).

2. M. Cardona, *Light Scattering in Solids 1*, Topics in Applied Physics (Springer Berlin Heidelberg, 2013).

3. M. Moskovits, Surface selection rules. *J. Chem. Phys.* **77**, 4408–4416 (1982).

4. T. Sander, C. T. Reindl, M. Giar, B. Eifert, M. Heinemann, C. Heiliger, P. J. Klar, Correlation of intrinsic point defects and the Raman modes of cuprous oxide. *Phys. Rev. B* **90**, 045203 (2014).

5. G. H. Loechelt, N. G. Cave, J. Menéndez, Measuring the tensor nature of stress in silicon using polarized off-axis Raman spectroscopy. *Appl. Phys. Lett.* **66**, 3639–3641 (1995).

6. K. Zhang, C. Bao, Q. Gu, X. Ren, H. Zhang, K. Deng, Y. Wu, Y. Li, J. Feng, S. Zhou, Raman signatures of inversion symmetry breaking and structural phase transition in type-II Weyl semimetal $MoTe_2$. *Nat. Commun.* **7**, 13552 (2016).

7. S. G. Drapcho, J. Kim, X. Hong, C. Jin, S. Shi, S. Tongay, J. Wu, F. Wang, Apparent breakdown of Raman selection rule at valley exciton resonances in monolayer MoS_2. *Phys. Rev. B* **95**, 165417 (2017).

8. X. Xi, L. Zhao, Z. Wang, H. Berger, L. Forró, J. Shan, K. F. Mak, Strongly enhanced charge-density-wave order in monolayer $NbSe_2$. *Nat. Nanotechnol.* **10**, 765–769 (2015).

9. B. Singh, G. McNamara, K. M. Kim, S. Siddique, S. D. Funni, W. Zhang, X. Luo, P. Sakrikar, E. M. Kenney, R. Singha, S. Alekseev, S. A. A. Ghorashi, T. J. Hicken, C. Baines, H. Luetkens, Y. Wang, V. M. Plisson, M. Geiwitz, C. A. Occhialini, R. Comin, M. J. Graf, L. Zhao, J. Cano, R. M. Fernandes, J. J. Cha, L. M. Schoop, K. S. Burch, Ferroaxial density wave from intertwined charge and orbital order in rare-earth tritellurides. *Nat. Phys.* **21**, 1578–1586 (2025).

10. Y. Zhang, X. Wu, B. B. Lyu, M. Wu, S. Zhao, J. Chen, M. Jia, C. Zhang, L. Wang, X. Wang, Y. Chen, J. Mei, T. Taniguchi, K. Watanabe, H. Yan, Q. Liu, L. Huang, Y. Zhao, M. Huang, Magnetic order-induced polarization anomaly of Raman scattering in 2D magnet CrI_3. *Nano Lett.* **20**, 729–734 (2020).

11. S. Li, E. Drueke, Z. Porter, W. Jin, Z. Lu, D. Smirnov, R. Merlin, S. D. Wilson, K. Sun, L. Zhao, Symmetry-resolved two-magnon excitations in a strong spin-orbit-coupled bilayer antiferromagnet. *Phys. Rev. Lett.* **125**, 087202 (2020).

12. B. Huang, J. Cenker, X. Zhang, E. L. Ray, T. Song, T. Taniguchi, K. Watanabe, M. A. McGuire, D. Xiao, X. Xu, Tuning inelastic light scattering via symmetry control in the two-dimensional magnet CrI_3. *Nat. Nanotechnol.* **15**, 212–216 (2020).

13. S. Liu, A. Granados del Águila, D. Bhowmick, C. K. Gan, T. Thu Ha do, M. A. Prosnikov, D. Sedmidubský, Z. Sofer, P. C. M. Christianen, P. Sengupta, Q. Xiong, Direct observation of magnon-phonon strong coupling in two-dimensional antiferromagnet at high magnetic fields. *Phys. Rev. Lett.* **127**, 097401 (2021).

14. T. P. Devereaux, R. Hackl, Inelastic light scattering from correlated electrons. *Rev. Mod. Phys.* **79**, 175–233 (2007).

15. J. Buhot, M. A. Méasson, Y. Gallais, M. Cazayous, A. Sacuto, G. Lapertot, D. Aoki, Symmetry of the excitations in the hidden order state of Uru_2Si_2. *Phys. Rev. Lett.* **113**, 266405 (2014).

16. H.-H. Kung, R. E. Baumbach, E. D. Bauer, V. K. Thorsmølle, W.-L. Zhang, K. Haule, J. A. Mydosh, G. Blumberg, Chirality density wave of the “hidden order” phase in Uru_2Si_2. *Science* **347**, 1339–1342 (2015).

17. E. M. Lacinska, M. Furman, J. Binder, I. Lutsyk, P. J. Kowalczyk, R. Stepniewski, A. Wysmolek, Raman optical activity of 1T-TaS_2. *Nano Lett.* **22**, 2835–2842 (2022).

18. Y.-X. Li, Y. Chen, L. Pan, S. Li, S.-B. Zhang, H.-Z. Lu, Exploration of altermagnetism in RuO_2. arXiv:2509.19932 [cond-mat.mes-hall] (2026).

19. H. Over, Surface chemistry of ruthenium dioxide in heterogeneous catalysis and electrocatalysis: From fundamental to applied research. *Chem. Rev.* **112**, 3356–3426 (2012).

20. T. Berlijn, P. C. Snijders, O. Delaire, H. D. Zhou, T. A. Maier, H. B. Cao, S. X. Chi, M. Matsuda, Y. Wang, M. R. Koehler, P. R. C. Kent, H. H. Weitering, Itinerant antiferromagnetism in RuO_2. *Phys. Rev. Lett.* **118**, 077201 (2017).

21. Z. H. Zhu, J. Strempfer, R. R. Rao, C. A. Occhialini, J. Pelliciari, Y. Choi, T. Kawaguchi, H. You, J. F. Mitchell, Y. Shao-Horn, R. Comin, Anomalous antiferromagnetism in metallic RuO_2 determined by resonant x-ray scattering. *Phys. Rev. Lett.* **122**, 017202 (2019).

22. L. S. Šmejkal, J. Sinova, T. Jungwirth, Emerging research landscape of altermagnetism. *Phys. Rev. X* **12**, 040501 (2022).

23. M. Hiraishi, H. Okabe, A. Koda, R. Kadono, T. Muroi, D. Hirai, Z. Hiroi, Nonmagnetic ground state in RuO_2 revealed by muon spin rotation. *Phys. Rev. Lett.* **132**, 166702 (2024).

24. P. Kebler, L. Garcia-Gassull, A. Suter, T. Prokscha, Z. Salman, D. Khalyavin, P. Manuel, F. Orlandi, I. I. Mazin, R. Valentı, S. Moser, Absence of magnetic order in RuO_2: Insights from μSR spectroscopy and neutron diffraction. *npj Spintronics* **2**, 50 (2024).

25. L. Kiefer, F. Wirth, A. Bertin, P. Becker, L. Bohatý, K. Schmalzl, A. Stunault, J. A. Rodríguez-Velamazan, O. Fabelo, M. Braden, Crystal structure and absence of magnetic order in single-crystalline RuO_2. *J. Phys. Condens. Matter* **37**, 135801 (2025).

26. J. Liu, J. Zhan, T. Li, J. Liu, S. Cheng, Y. Shi, L. Deng, M. Zhang, C. Li, J. Ding, Q. Jiang, M. Ye, Z. Liu, Z. Jiang, S. Wang, Q. Li, Y. Xie, Y. Wang, S. Qiao, J. Wen, Y. Sun, D. Shen, Absence of altermagnetic spin splitting character in rutile oxide RuO_2. *Phys. Rev. Lett.* **133**, 176401 (2024).

27. A. Smolyanyuk, I. I. Mazin, L. Garcia-Gassull, R. Valentí, Fragility of the magnetic order in the prototypical altermagnet RuO_2. *Phys. Rev. B* **109**, 134424 (2024).

28. Z. Qian, Y. Yang, S. Liu, C. Wu, Fragile unconventional magnetism in RuO_2 by proximity to Landau-Pomeranchuk instability. *Phys. Rev. B* **111**, 174425 (2025).

29. V. Jovic, R. J. Koch, S. K. Panda, H. Berger, P. Bugnon, A. Magrez, K. E. Smith, S. Biermann, C. Jozwiak, A. Bostwick, E. Rotenberg, S. Moser, Dirac nodal lines and flat-band surface state in the functional oxide RuO_2. *Phys. Rev. B* **98**, 241101 (2018).

30. K.-H. Ahn, A. Hariki, K.-W. Lee, J. Kuneš, Antiferromagnetism in RuO_2 as *d*-wave Pomeranchuk instability. *Phys. Rev. B* **99**, 184432 (2019).

31. L. Šmejkal, R. González-Hernández, T. Jungwirth, J. Sinova, Crystal time-reversal symmetry breaking and spontaneous Hall effect in collinear antiferromagnets. *Sci. Adv.* **6**, eaaz8809 (2020).

32. A. Bose, N. J. Schreiber, R. Jain, D. F. Shao, H. P. Nair, J. Sun, X. S. Zhang, D. A. Muller, E. Y. Tsymbal, D. G. Schlom, D. C. Ralph, Tilted spin current generated by the collinear antiferromagnet ruthenium dioxide. *Nat. Electron.* **5**, 267–274 (2022).

33. O. Fedchenko, J. Minár, A. Akashdeep, S. W. D'Souza, D. Vasilyev, O. Tkach, L. Odenbreit, Q. Nguyen, D. Kutnyakhov, N. Wind, L. Wenthaus, M. Scholz, K. Rossnagel, M. Hoesch, M. Aeschlimann, B. Stadtmüller, M. Kläui, G. Schönhense, T. Jungwirth, A. B. Hellenes, G. Jakob, L. Šmejkal, J. Sinova, H. J. Elmers, Observation of time-reversal symmetry breaking in the band structure of altermagnetic RuO_2. *Sci. Adv.* **10**, eadj4883 (2024).

34. X. Feng, H. Bai, X. Fan, M. Guo, Z. Zhang, G. Chai, T. Wang, D. Xue, C. Song, X. Fan, incommensurate spin density wave in antiferromagnetic RuO_2 evinced by abnormal spin splitting torque. *Phys. Rev. Lett.* **132**, 086701 (2024).

35. X. He, L. Cai, Y. Liang, F. Yang, L. Yi, C. Pan, W. Wang, X. Qiu, Z. Xia, L. Pan, Magnetic-field dependent néel vector perturbation and its magneto-transport properties of RuO_2 epitaxial films. *Adv. Funct. Mater.* **36**, e20771 (2026).

36. H. Chen, Z. A. Wang, P. Qin, Z. Meng, X. Zhou, X. Wang, L. Liu, G. Zhao, Z. Duan, T. Zhang, J. Liu, D. F. Shao, C. Jiang, Z. Liu, Spin-splitting magnetoresistance in altermagnetic RuO_2 thin films. *Adv. Mater.* **37**, 2507764 (2025).

37. D. Cheng, J. Li, S. Luo, Z. Chen, X. Li, Rotational anisotropy Raman spectrometer for high-sensitivity crystallographic symmetry analysis. *Laser Photonics Rev.* e71658 (2026).

38. Y. S. Huang, F. H. Pollak, Raman investigation of rutile RuO_2. *Solid State Commun.* **43**, 921–924 (1982).

39. S. S. Rosenblum, W. H. Weber, B. L. Chamberland, Raman-scattering observation of the rutile-to-$CaCl_2$ phase transition in RuO_2. *Phys. Rev. B* **56**, 529–533 (1997).

40. Y. M. Chen, A. Korotcov, H. P. Hsu, Y. S. Huang, D. S. Tsai, Raman scattering characterization of well-aligned RuO_2 nanocrystals grown on sapphire substrates. *New J. Phys.* **9**, 130 (2007).

41. L. D. Barron, Magnetic vibrational optical activity in the resonance Raman spectrum of ferrocytochrome c. *Nature* **257**, 372–374 (1975).

42. L. A. Nafie, J. C. Lindon, G. E. Tranter, D. W. Koppenaal, "Raman optical activity, theory," in *Encyclopedia of Spectroscopy and Spectrometry* (Academic Press, ed. 3, 2017), pp. 891–899.

43. J. Thapa, B. Liu, S. D. Woodruff, B. T. Chorpening, M. P. Buric, Raman scattering in single-crystal sapphire at elevated temperatures. *Appl. Optics* **56**, 8598–8606 (2017).

44. K. J. Kapcia, M. Reedyk, M. Hajialamdari, A. Ptok, P. Piekarz, A. Schulz, F. S. Razavi, R. K. Kremer, A. M. Oleś, Discovery of a low-temperature orthorhombic phase of the $Cd_2Re_2O_7$ superconductor. *Phys. Rev. Res.* **2**, 033108 (2020).

45. A. de la Torre, K. L. Seyler, L. Zhao, S. Di Matteo, M. S. Scheurer, Y. Li, B. Yu, M. Greven, S. Sachdev, M. R. Norman, D. Hsieh, Mirror symmetry breaking in a model insulating cuprate. *Nat. Phys.* **17**, 777–781 (2021).

46. K. R. Hoffman, W. M. Yen, D. J. Lockwood, P. E. Sulewski, Birefringence-induced vibrational Raman and Rayleigh optical activity in uniaxial crystals. *Phys. Rev. B* **49**, 182–188 (1994).

47. M. Wenzel, E. Uykur, S. Rößler, M. Schmidt, O. Janson, A. Tiwari, M. Dressel, A. A. Tsirlin, Fermi-liquid behavior of nonaltermagnetic RuO_2. *Phys. Rev. B* **111**, L041115 (2025).

48. I. Gregora, "Raman scattering," in *International Tables for Crystallography* (John Wiley & Sons Ltd., 2013), pp. 334–348; https://onlinelibrary.wiley.com/doi/abs/10.1107/97809553602060000913.

49. R. M. Martin, T. C. Damen, Breakdown of selection rules in resonance Raman scattering. *Phys. Rev. Lett.* **26**, 86–88 (1971).

50. F.-c. Liu, A. D. Buckingham, Antisymmetric resonant vibrational Raman scattering. *Chem. Phys. Lett.* **207**, 325–331 (1993).

51. Q.-H. Tan, Y. J. Sun, X. L. Liu, K. X. Xu, Y. F. Gao, S. L. Ren, P. H. Tan, J. Zhang, Breakdown of Raman selection rules by Fröhlich interaction in few-layer WS_2. *Nano Res.* **14**, 239–244 (2021).

52. E. T. Heyen, S. N. Rashkeev, I. I. Mazin, O. K. Andersen, R. Liu, M. Cardona, O. Jepsen, Resonant Raman scattering in $YBa_2Cu_3O_7$: Band theory and experiment. *Phys. Rev. Lett.* **65**, 3048–3051 (1990).

53. M. Dresselhaus, G. Dresselhaus, A. Jorio, *Group Theory: Application to the Physics of Condensed Matter* (Springer Berlin Heidelberg, 2007).

54. D. Saparov, B. Xiong, Y. Ren, Q. Niu, Lattice dynamics with molecular Berry curvature: Chiral optical phonons. *Phys. Rev. B* **105**, 064303 (2022).

55. C. A. Mead, D. G. Truhlar, On the determination of Born-Oppenheimer nuclear motion wave functions including complications due to conical intersections and identical nuclei. *J. Chem. Phys.* **70**, 2284–2296 (1979).

56. S. Coh, Classification of materials with phonon angular momentum and microscopic origin of angular momentum. *Phys. Rev. B* **108**, 134307 (2023).

57. J. Bonini, S. Ren, D. Vanderbilt, M. Stengel, C. E. Dreyer, S. Coh, Frequency splitting of chiral phonons from broken time-reversal symmetry in CrI_3. *Phys. Rev. Lett.* **130**, 086701 (2023).

58. S. Chaudhary, D. M. Juraschek, M. Rodriguez-Vega, G. A. Fiete, Giant effective magnetic moments of chiral phonons from orbit-lattice coupling. *Phys. Rev. B* **110**, 094401 (2024).

59. M. Che, J. Liang, Y. Cui, H. Li, B. Lu, W. Sang, X. Li, X. Dong, L. Zhao, S. Zhang, T. Sun, W. Jiang, E. Liu, F. Jin, T. Zhang, L. Yang, Magnetic order induced chiral phonons in a ferromagnetic Weyl semimetal. *Phys. Rev. Lett.* **134**, 196906 (2025).

60. L. Zhang, J. Ren, J.-S. Wang, B. Li, Topological nature of the phonon hall effect. *Phys. Rev. Lett.* **105**, 225901 (2010).

61. K. Sun, Z. Gao, J.-S. Wang, Current-induced phonon Hall effect. *Phys. Rev. B* **102**, 134311 (2020).

62. E. Torun, C. M. Fang, G. A. de Wijs, R. A. de Groot, Role of magnetism in catalysis: RuO_2 (110) surface. *J. Phys. Chem. C* **117**, 6353–6357 (2013).

63. D. Q. Ho, D. Q. To, R. Hu, G. W. Bryant, A. Janotti, Symmetry-breaking induced surface magnetization in nonmagnetic RuO_2. *Phys. Rev. Mater.* **9**, 094406 (2025).

64. W. Witczak-Krempa, G. Chen, Y. B. Kim, L. Balents, Correlated quantum phenomena in the strong spin-orbit regime. *Annu. Rev. Condens. Matter Phys.* **5**, 57–82 (2014).

65. H. Ning, O. Mehio, X. Li, M. Buchhold, M. Driesse, H. Zhao, G. Cao, D. Hsieh, A coherent phonon-induced hidden quadrupolar ordered state in Ca_2RuO_4. *Nat. Commun.* **14**, 8258 (2023).

66. H. Watanabe, R. Oiwa, R. Arita, Dual-circular Raman optical activity of axial multipolar order. arXiv:2507.09237 [physics.optics] (2025).

67. S. W. Lovesey, D. D. Khalyavin, G. van der Laan, Magnetic structure of RuO_2 in view of altermagnetism. *Phys. Rev. B* **108**, L121103 (2023).

68. S. Bhowal, N. A. Spaldin, Ferroically ordered magnetic octupoles in *d*-wave altermagnets. *Phys. Rev. X* **14**, 011019 (2024).

69. C. M. Varma, Non-Fermi-liquid states and pairing instability of a general model of copper oxide metals. *Phys. Rev. B* **55**, 14554–14580 (1997).

70. P. Bourges, D. Bounoua, Y. Sidis, Loop currents in quantum matter. *C. R. Phys.* **22**, 7–31 (2021).

71. G. Palle, R. Ojajärvi, R. M. Fernandes, J. Schmalian, Superconductivity due to fluctuating loop currents. *Sci. Adv.* **10**, eadn3662 (2024).

72. R. Fittipaldi, R. Hartmann, M. T. Mercaldo, S. Komori, A. Bjørlig, W. Kyung, Y. Yasui, T. Miyoshi, L. A. B. Olde Olthof, C. M. Palomares Garcia, V. Granata, I. Keren, W. Higemoto, A. Suter, T. Prokscha, A. Romano, C. Noce, C. Kim, Y. Maeno, E. Scheer, B. Kalisky, J. W. A. Robinson, M. Cuoco, Z. Salman, A. Vecchione, A. di Bernardo, Unveiling unconventional magnetism at the surface of Sr_2RuO_4. *Nat. Commun.* **12**, 5792 (2021).

73. M. A. Pimenta, G. C. Resende, H. B. Ribeiro, B. R. Carvalho, Polarized Raman spectroscopy in low-symmetry 2D materials: Angle-resolved experiments and complex number tensor elements. *Phys. Chem. Chem. Phys.* **23**, 27103–27123 (2021).

74. J. W. Harter, L. Niu, A. J. Woss, D. Hsieh, High-speed measurement of rotational anisotropy nonlinear optical harmonic generation using position-sensitive detection. *Opt. Lett.* **40**, 4671–4674 (2015).

75. D. J. Lockwood, K. R. Hoffman, W. M. Yen, Magnetic Raman optical activity in FeF_2. *J. Lumin.* **100**, 147–154 (2002).

76. L. D. Barron, M. P. Bogaard, A. D. Buckingham, Raman scattering of circularly polarized light by optically active molecules. *J. Am. Chem. Soc.* **95**, 603–605 (1973).

77. F. Wu, J. Zhou, S. Bao, L. Li, J. Wen, Y. Wan, Q. Zhang, Magnetic switching of phonon angular momentum in a ferrimagnetic insulator. *Phys. Rev. Lett.* **134**, 236701 (2025).

78. S. P. S. Porto, J. A. Giordmaine, T. C. Damen, Depolarization of Raman scattering in calcite. *Phys. Rev.* **147**, 608–611 (1966).

79. Y. Tatsumi, T. Kaneko, R. Saito, Conservation law of angular momentum in helicity-dependent Raman and Rayleigh scattering. *Phys. Rev. B* **97**, 195444 (2018).

80. A. K. Goel, G. Skorinko, F. H. Pollak, Optical properties of single-crystal rutile RuO_2 and IrO_2 in the range 0.5 to 9.5 eV. *Phys. Rev. B* **24**, 7342–7350 (1981).

81. Y. Ze-Jin, G. Yun-Dong, L. Jin, L. Jin-Chao, D. Wei, C. Xin-Lu, Y. Xiang-Dong, Electronic structure and optical properties of rutile RuO_2 from first principles. *Chin. Phys. B* **19**, 077102 (2010).

82. J. Lekner, Reflection and refraction by uniaxial crystals. *J. Phys. Condens. Matter* **3**, 6121–6133 (1991).

83. G. Kresse, J. Furthmüller, Efficiency of ab-initio total energy calculations for metals and semiconductors using a plane-wave basis set. *Comput. Mater. Sci.* **6**, 15–50 (1996).

84. G. Kresse, J. Furthmüller, Efficient iterative schemes for ab initio total-energy calculations using a plane-wave basis set. *Phys. Rev. B* **54**, 11169–11186 (1996).

85. J. P. Perdew, K. Burke, M. Ernzerhof, Generalized gradient approximation made simple. *Phys. Rev. Lett.* **77**, 3865–3868 (1996).

86. S. L. Dudarev, G. A. Botton, S. Y. Savrasov, C. J. Humphreys, A. P. Sutton, Electron-energy-loss spectra and the structural stability of nickel oxide: An LSDA+U study. *Phys. Rev. B* **57**, 1505–1509 (1998).

87. X. Gonze, C. Lee, Dynamical matrices, Born effective charges, dielectric permittivity tensors, and interatomic force constants from density-functional perturbation theory. *Phys. Rev. B* **55**, 10355–10368 (1997).

88. S. Baroni, S. de Gironcoli, A. Dal Corso, P. Giannozzi, Phonons and related crystal properties from density-functional perturbation theory. *Rev. Mod. Phys.* **73**, 515–562 (2001).

89. P. Umari, A. Pasquarello, A. Dal Corso, Raman scattering intensities in α-quartz: A first-principles investigation. *Phys. Rev. B* **63**, 094305 (2001).

90. P. Umari, A. Pasquarello, Infrared and Raman spectra of disordered materials from first principles. *Diamond Relat. Mater.* **14**, 1255–1261 (2005).

91. A. Calzolari, M. B. Nardelli, Dielectric properties and Raman spectra of ZnO from a first principles finite-differences/finite-fields approach. *Sci. Rep.* **3**, 2999 (2013).

92. Y. Gillet, M. Giantomassi, X. Gonze, First-principles study of excitonic effects in Raman intensities. *Phys. Rev. B* **88**, 094305 (2013).

93. M. Bagheri, H.-P. Komsa, High-throughput computation of Raman spectra from first principles. *Sci. Data* **10**, 80 (2023).

94. M. Gajdoš, K. Hummer, G. Kresse, J. Furthmüller, F. Bechstedt, Linear optical properties in the projector-augmented wave methodology. *Phys. Rev. B* **73**, 045112 (2006).

95. K. Sun, Z. Gao, J.-S. Wang, Phonon Hall effect with first-principles calculations. *Phys. Rev. B* **103**, 214301 (2021).

96. L. Zhang, Q. Niu, Angular momentum of phonons and the Einstein–de Haas effect. *Phys. Rev. Lett.* **112**, 085503 (2014).

97. T. Saito, K. Misaki, H. Ishizuka, N. Nagaosa, Berry phase of phonons and thermal Hall effect in nonmagnetic insulators. *Phys. Rev. Lett.* **123**, 255901 (2019).

98. S. M. C. Parlak, S. Ghosh, I. Garate, Detection of phonon helicity in nonchiral crystals with Raman scattering. *Phys. Rev. B* **107**, 104308 (2023).

99. J. J. Nakane, H. Kohno, Angular momentum of phonons and its application to single-spin relaxation. *Phys. Rev. B* **97**, 174403 (2018).

100. C. S. Knee, J. Holmlund, J. Andreasson, M. Käll, S. G. Eriksson, L. Börjesson, Order-disorder-order phase transitions in the pyrochlore superconductor $Cd_2Re_2O_7$. *Phys. Rev. B* **71**, 214518 (2005).

101. Y. S. Huang, S. S. Lin, C. R. Huang, M. C. Lee, T. E. Dann, F. Z. Chien, Raman spectrum of IrO_2. *Solid State Commun.* **70**, 517–522 (1989).

102. K.-P. Bohnen, R. Heid, O. P. Seaman, B. Renker, P. Adelmann, H. Schober, Lattice dynamics of RuO_2: Theory and experiment. *Phys. Rev. B* **75**, 092301 (2007).

103. P. R. Raghuvanshi, T. Berlijn, D. S. Parker, S. Wang, M. E. Manley, R. P. Hermann, L. Lindsay, V. R. Cooper, Altermagnetic behavior in OsO_2: Parallels with RuO_2. *Phys. Rev. Mater.* **9**, 034407 (2025).

104. G. Yumnam, P. R. Raghuvanshi, J. D. Budai, L. Bocklage, D. Abernathy, Y. Cheng, A. H. Said, I. I. Mazin, H. Zhou, B. A. Frandsen, D. S. Parker, L. R. Lindsay, V. R. Cooper, M. E. Manley, R. P. Hermann, Constraints on magnetism and correlations in RuO_2 from lattice dynamics and Mössbauer spectroscopy. *Cell Rep. Phys. Sci.* **6**, 102852 (2025).
